# Development, validation of a GC–MS method for the simultaneous measurement of amino acids, their PTM metabolites and AGEs in human urine, and application to the bi-ethnic ASOS study with special emphasis to lysine

**DOI:** 10.1007/s00726-021-03031-6

**Published:** 2021-07-12

**Authors:** Svetlana Baskal, Alexander Bollenbach, Catharina Mels, Ruan Kruger, Dimitrios Tsikas

**Affiliations:** 1 Core Unit Proteomics, Institute of Toxicology, Hannover Medical School, Carl-Neuberg-Strasse 1, 30625 Hannover, Germany; 2 Hypertension in Africa Research Team (HART), North-West University, Potchefstroom, South Africa; 3 MRC Research Unit for Hypertension and Cardiovascular Disease, North-West University, Potchefstroom, South Africa

**Keywords:** AGEs, Amino acids, ASOS, Derivatization, Deuterium, GC–MS, Post-translational modification (PTM), Quantification

## Abstract

A gas chromatography-mass spectrometry (GC–MS) method was developed and validated in relevant concentration ranges for the simultaneous measurement of l-lysine (Lys, L) and its *N*^ε^- and *N*^α^-methylated (M), *N*^ε^- and *N*^α^-acetylated (Ac), *N*^ε^-carboxymethylated (CM) and *N*^ε^-carboxyethylated (CE) metabolites in human urine. Analyzed Lys metabolites were the post-translational modification (PTM) products *N*^ε^-mono-, di- and trimethyllsine, *N*^ε^-MML, *N*^ε^-DML, *N*^ε^-TML, respectively, *N*^α^-ML, *N*^ε^-AcL, *N*^α^-AcL, and its advanced glycation end-products (AGEs) *N*^ε^-CML, *N*^ε^-CM-[2,4,4-^2^H_3_]Lys (d_3_-CML), *N*^ε^-CEL and furosine. AGEs of arginine (Arg) and cysteine (Cys) were also analyzed. De novo synthesized trideutero-methyl esters (R-COOCD_3_) from unlabelled amino acids and derivatives were used as internal standards. Native urine samples (10 µL aliquots) were evaporated to dryness under a stream of nitrogen. Analytes were esterified using 2 M HCl in methanol (60 min, 80 °C) and subsequently amidated by pentafluoropropionic anhydride in ethyl acetate (30 min, 65 °C). The generated methyl ester-pentafluoropropionyl (Me-PFP) derivatives were reconstituted in borate buffer and extracted immediately with toluene. GC–MS analyses were performed by split-less injection of 1-µL aliquots, oven-programmed separation and negative-ion chemical ionization (NICI). Mass spectra were generated in the scan mode (range, *m*/*z* 50–1000). Quantification was performed in the selected-ion monitoring (SIM) mode using a dwell time of 50 or 100 ms for each ion. The GC–MS method was suitable for the measurement of Lys and all of its metabolites, except for the quaternary ammonium cation *N*^ε^-TML. The Me-PFP derivatives of Lys, Arg and Cys and its metabolites eluted in the retention time window of 9 to 14 min. The derivatization of *N*^ε^-CML, d_3_-CML and *N*^ε^-CEL was accompanied by partial *N*^ε^-decarboxylation and formation of the Me-PFP Lys derivative. The lowest derivatization yield was observed for *N*^ε^-DML, indicating a major role of the *N*^ε^-DML group in Lys derivatization. The GC–MS method enables precise (relative standard deviation, RSD < 20%) and accurate (bias, < ± 20%) simultaneous measurement of 33 analytes in human urine in relevant concentration ranges. We used the method to measure the urinary excretion rates of Lys and its PTM metabolites and AGEs in healthy black (*n* = 39) and white (*n* = 41) boys of the Arterial Stiffness in Offspring Study (ASOS). No remarkable differences were found indicating no ethnic-related differences in PTM metabolites and AGEs except for *N*^ε^-monomethyllysine and *S*-(2-carboxymethylcysteine).

**Supplementary Information:**

The online version contains supplementary material available at 10.1007/s00726-021-03031-6.

## Introduction

**Fig. 1:**
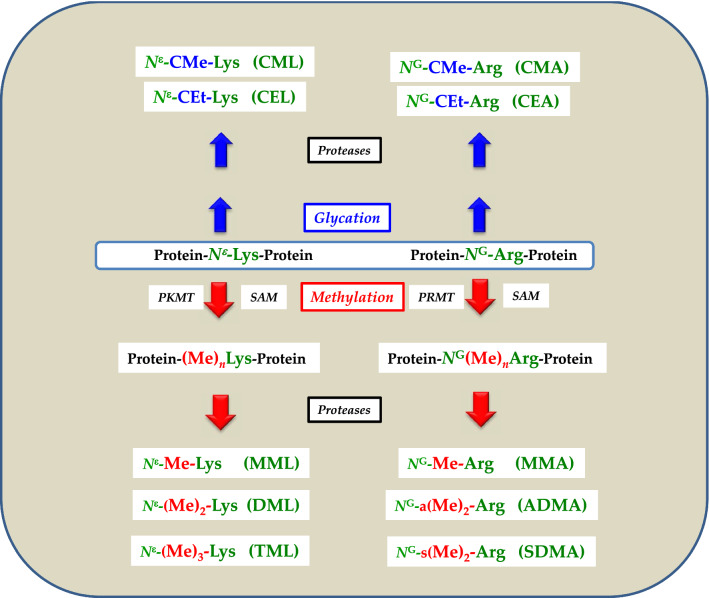
Simplified schematic of two post-translational modifications, i.e., glycation and methylation of the *N*-terminal *N*-epsilon (*N*^ε^) amine group of l-lysine (L) and of the terminal *N*-guanidine (*N*^G^) amine group(s) of l-arginine (A) residues in proteins. *CM* carboxymethyl, *CE* carboxyethyl, *PKMT* protein-lysine methyltransferase, *PRMT* protein-arginine methyltransferase, *SAM* *S*-adenosylmethionine, *n* number of methyl groups, *a* asymmetric, *s* symmetric. For a detailed description of reactions and metabolites, see the text

Proteinogenic amino acid residues undergo multiple post-translational modifications (PTM) including glycation. Well-investigated PTM include the methylation of the terminal ε-amine (*N*^ε^) group of l-lysine and the terminal guanidine (*N*^G^) group of l-arginine (Fig. 1). These reactions are catalyzed by protein-lysine methyl transferases (PKMT, EC 2.1.1.43) and protein-arginine methyl transferases (PRMT, EC 2.1.1.125), respectively. Both enzyme families use *S*-adenosylmethionine (SAM) as the common methyl group (Me) donor. Arg, Lys and l-cysteine (Cys) also undergo chemical glycation on *N*^G^, *N*^ε^ and S, respectively. Proteolysis of glycated and methylated proteins releases the low-molecular-mass (LMM) glycated and methylated amino acids (Fig. 1). The LMM advanced glycation end-products (AGEs) and methylated amino acids circulate in the blood and are finally excreted in the urine in their native forms or as metabolites.

The biological functions of PTM proteins and their LMM metabolites are largely unknown. The concentration of circulating and excretory LMM metabolites, including the AGEs, is commonly used as surrogate of the extent of PTM in generally unknown proteins in health and disease. In particular, AGEs are widely used as markers for carbohydrate metabolism and protein denaturation (Nagai et al. 2014). The *N*^G^-methylated Arg metabolites monomethylarginine (MMA), asymmetric dimethylarginine (ADMA) and symmetric dimethylarginine (SDMA) are physiological inhibitors of nitric oxide synthase (NOS; EC 1.14.13.39) (Tsikas et al. 2000). NOS isoforms oxidize the *N*^G^ group of free l-arginine to l-citrulline and nitric oxide (NO) which plays multiple physiological roles. Circulating and excretory ADMA and SDMA were found to be associated with renal and cardiovascular disease, inflammation and cancer (Oliva-Damaso et al. 2019; Said et al. 2019; Dowsett et al. 2020; Samuel et al. 2021), yet not necessarily in their function as NOS activity inhibitors (Tsikas et al. 2018). Analogous to Arg, proteinic Lys is also methylated on its *N*^ε^ group to finally form *N*^ε^-monomethyllysine (MML), *N*^ε^-dimethyllysine (DML) and *N*^ε^-trimethyllysine (TML). In contrast to MMA, ADMA and SDMA, the biological significance of the Lys metabolites MML, DML and TML, especially in the renal and cardiovascular systems, is far less known (Schwedhelm et al. 2021). AGEs seem to play a significant role in cardiovascular disease including heart failure with preserved and reduced ejection fraction (Willemsen et al. 2012; Paulus and Dal Canto 2018; Baskal et al. 2021a). For instance, higher plasma CML concentrations were significantly and independently associated with higher risk for mortality (Willemsen et al. 2012). AGEs are considered to exert their biological activity via the soluble form of their receptor, i.e., sRAGE. Selected circulating and excretory AGEs have been reported to differ in healthy and diseased children (Petrovič et al. 2005; Dittrich et al. 2006) and in adults (Maasen et al. 2019).

Native and derivatized biological amino acids, their PTM metabolites and AGEs are measured by many different liquid chromatographic (LC), gas chromatographic (GC) and electrophoretic techniques coupled with various detection techniques. They include visible, ultraviolet absorbance and fluorescence detection, as well as mass spectrometry (MS) and tandem mass spectrometry (MS/MS) as separation techniques, such as GC–MS and LC–MS/MS (Martens-Lobenhoffer and Bode-Böger 2014; Hušek et al. 2016; Rabbani and Thornalley 2020; Gałęzowska et al. 2021).

In our group, we use gas chromatography-mass spectrometry (GC–MS) methods for the quantitative determination of endogenous substances including amino acids and their metabolites in biological samples. GC–MS is useful for the reliable quantitation of amino acids and their metabolites. For instance, Arg and its inorganic metabolites nitrite and nitrate are measured by GC–MS and used as surrogates for NO (Tsikas 2000; Hanff et al. 2019). The organic Arg metabolites ADMA and SDMA (Hanff et al. 2019) are measured by GC–MS and used as surrogates of asymmetric and symmetric *N*^G^ methylation of Arg, respectively (Bollenbach et al. 2018). GC–MS was also found to be useful to measure circulating and excretory l-homoarginine (hArg) and guanidinoacetate (GAA) from other Arg-involving pathways (Tsikas 2009a; Kayacelebi et al. 2014; Tsikas and Wu 2015; Hanff et al. 2016, 2019).

GC–MS analysis of amino acids requires chemical derivatization (Hušek and Macek, 1975; Hušek et al. 2016; Baskal et al. 2021a, b). Our GC–MS methods for amino acids involve a two-step derivatization procedure under strong acidic (2 M HCl) and thermal conditions (80 °C and 65 °C). Under such conditions, the carbamoyl amino acids citrulline (Cit), Gln and Asp are converted into ornithine (Orn), Glu and Asp, respectively (Baskal et al. 2021a, b). Whether these GC–MS methods are also useful for the quantitative determination of the AGEs of Lys, Arg and Cys in biological samples have not been investigated thus far. As AGEs consist of an amino acid residue and a glycation residue (possibly derived from glyoxal, methylglyoxal or succinic acid), their derivatization and GC–MS analysis may lead to decomposition and release of the parent amino acid and the *N*-terminal glycyl residue.

The bi-ethnic study ASOS, the Arterial Stiffness in Offspring study, was originally conducted to investigate the link of urinary metabolites with premature arterial stiffness and the early detection and identification of cardiovascular disease and hypertension development in black and white populations from South Africa (Erasmus et al. 2019). The effects of PTM metabolites and AGEs on the arterial wall are considered to be direct or mediated by chronic inflammation and oxidative stress, and are assumed to result in arterial stiffening and decreased vascular compliance (Zanoli et al. 2019). Previous results from the ASOS study revealed ethnic-dependent differences in healthy black and white boys with respect to ADMA and SDMA, the Arg PTM metabolites, as well as with respect to nitrite and nitrate, the metabolites of free Arg-derived NO (Bollenbach et al. 2018, 2020a; Erasmus et al. 2019; Craig et al. 2020). The aim of the study was to develop and validate a stable-isotope dilution GC–MS method for the simultaneous quantitative determination of several PTM metabolites and AGEs alongside their precursor amino acids in human urine for use in epidemiologic and clinical settings.

Thus, we investigated systematically the GC–MS properties of *N*^ε^-methylated metabolites of Lys and its AGEs, as well as of commonly measured AGEs of Arg and Cys. After thorough validation in human urine, the GC–MS method was applied in the ASOS study to investigate potential ethnic-dependent differences in PTM and AGEs of Lys, Arg and Cys. To our knowledge, this is the first study to report on the GC–MS method and its application to urinary excretion of AGEs derived from Lys, Arg and Cys in healthy black and whites boys from South Africa.

## Materials and methods

### Chemicals, materials and reagents

l-Lysine, *N*^ε^-methyl-l-lysine, *N*^ε^,*N*^ε^-dimethyl-l-lysine, *N*^ε^,*N*^ε^,*N*^ε^-trimethyl-l-lysine, *N*^α^-methyl-l-lysine, *N*^α^-acetyl-l-lysine, *N*^ε^-acetyl-l-lysine (chemical purity, 95 to 98%) and d,l-5-hydroxy-l-lysine hydrochloride salts, *N*^G^-methyl-l-arginine acetate salt, *S*-(2-succinyl)-l-cysteine (chemical purity by TLC, 98%) and *S*-carboxymethyl-l-cysteine (chemical purity, 98%) were obtained from Sigma-Aldrich. *N*^ε^-(1-Carboxymethyl)-l-lysine (chemical purity by TLC, 95%), *N*^ε^-(1-carboxymethyl)-l-[2,4,4-^2^H_3_]-lysine (chemical purity by TLC, 98%; isotopic purity, 99% ^2^H) and *N*^ε^-(1-carboxyethyl)-l-lysine (chemical purity, 95%) were purchased from Cayman. *N*^G^-Carboxymethyl-l-arginine and *N*^G^-carboxyethyl-l-arginine (chemical purity, 98%) were obtained from Iris Biotech GmbH (Marktredwitz, Germany). Furosine hydrochloride was purchased from Carbosynth (Eching, Germany) and *S*-(2-carboxyethyl)-l-cysteine (chemical purity, 98%) from TCI (Eschborn, Germany). Tetradeuterated methanol (CD_3_OD, 99% at ^2^H) and pentafluoropropionic anhydride were supplied by Sigma-Aldrich (Steinheim, Germany). Methanol was obtained from Chemsolute (Renningen, Germany). Hydrochloric acid (37 wt%) was purchased from Baker (Deventer, The Netherlands). Ethyl acetate was obtained from Merck (Darmstadt, Germany). Glassware for GC–MS (1.5-mL autosampler vials and 0.2-mL microvials) and the fused-silica capillary column Optima 17 (15 m × 0.25 mm I.D., 0.25-µm film thickness) were purchased from Macherey–Nagel (Düren, Germany).

Separate stock solutions of amino acids were prepared by dissolving accurately weighed amounts of commercially available unlabelled and stable-isotope labelled amino acids and metabolites in deionized water. Stock solutions were diluted with deionized water as appropriate.

For the preparation of unlabelled methyl esters and deuterium-labelled methyl esters of amino acids, two derivatization reagents were prepared. The esterification reagent 2 M HCl/CH_3_OH was prepared by adding slowly under gentle mixing to 80 mL ice-cold CH_3_OH and 16 mL of 37 wt% HCl. Analogous to 80 mL ice-cold CD_3_OD was added 16 mL of 37 wt% HCl slowly under gentle mixing to obtain the esterification reagent 2 M HCl/CD_3_OD. The concentration of HCl in these methanolic solutions was each 2 M. The acylation reagent PFPA/EA was prepared daily by diluting pure PFPA in ethyl acetate (EA) (1:4, v/v).

### Derivatization procedures for urinary amino acids and their metabolites

Methyl esters (Me) of the amino acids and their metabolites analyzed in the present study were prepared as follows. Urine (10 µL aliquots) was evaporated to dryness under a stream of nitrogen, the residues were reconstituted in 100 µL aliquots of a 2 M HCl/MeOH solution and the vials were tightly sealed. Esterification was performed by heating the samples for 60 min at 80 °C. After cooling to room temperature, urine extracts were spiked with 10 µL aliquots of the newly synthesized trideutero-methyl esters, i.e., the internal standard mixture, to reach relevant concentrations with respect to human urine, and the solvents were evaporated to dryness under a stream of nitrogen. Then, 100 µL aliquots of the PFPA/EA were added, the glass vials were tightly sealed and heated for 30 min at 65 °C to prepare *N*-pentafluoropropionic (PFP) amides of the methyl esters. After cooling to room temperature, solvents and reagents were evaporated to dryness under a stream of nitrogen. Subsequently, residues were treated first with 200 µL aliquots of 400 mM borate buffer, pH 8.5, and immediately thereafter with 200 µL aliquots of toluene, followed by immediate vortex-mixing for 60 s, and centrifugation (4000×*g*, 5 min, 18 °C). This step was used to eliminate acidic components and to extract the Me-PFP derivatives into toluene. Aliquots (150 µL) of the upper organic phase were transferred into autosampler glass vials equipped with micro inserts, the vials were sealed and the samples were subjected to GC–MS analysis.

### Generation of GC–MS spectra of derivatives of amino acids and their metabolites

Mass spectra were obtained after derivatization of two aliquots containing 5 nmol of each synthetic amino acid with 2 M HCl/CH_3_OH and 2 M HCl/CD_3_OD, respectively, followed by derivatization with PFPA/EA as described above. Commercially available amino acids labelled with stable isotopes, such as *N*^ε^-(1-carboxymethyl)-l-[2,4,4-^2^H_3_]-lysine (d_3_-CML), were derivatized with 2 M HCl/CH_3_OH followed by PFPA/EA. Derivatives were extracted with toluene (1 mL) as described above and 1 µL aliquots containing 5 pmol of each analyte (assuming quantitative yield) were injected in the split-less mode and mass spectra were generated in the scan mode after negative-ion chemical ionization (NICI).

### Quantitative analyses of amino acids and their metabolites

In quantitative analyses in human urine samples, the concentration of amino acids and their metabolites was determined by GC–MS in the selected-ion monitoring (SIM) mode using each one mass fragment for the unlabelled amino acids and the corresponding mass fragment for the stable-isotope labelled amino acids, which served as internal standards. A detailed description of the quantitative analyses is reported in the “Results” section. Quantification was performed by multiplying the peak area ratio (PAR) of the endogenous amino acid to the internal standard by the known concentration of the respective internal standard.

### Method validation in human urine

Method validation was performed using 10 µL aliquots of a pooled urine donated by a healthy volunteer. The concentration ranges of the amino acids and their metabolites added to the urine samples correspond to reported concentration ranges for the individual analytes in urine of healthy and diseased humans (Tsikas 2009b). The internal standard mixture for the urine samples was treated separately, yet in the same way. All analyses were performed in triplicate for each analyte concentration. Each 10 µL aliquot of the spiked and non-spiked urine samples was transferred into auto-sampler glass vials, solvents were evaporated to dryness under a stream of nitrogen, and derivatization was performed as described above. Detailed information of the method validation is reported in the “Results” section.

### Subjects: the Arterial Stiffness in Offspring Study (ASOS)

We applied the current method to the quantification of amino acids, their PTM metabolites and AGEs in 10 µL aliquots of spot urine samples of 39 healthy black boys and 41 healthy white boys (aged 6–8 years) collected in a previous study after approval by the local Ethics Committee (Mokwatsi et al. 2017). Ethical statement: participants were fully informed about the objective of the study (written informed consent and assent were obtained from all participants included in the study). All procedures performed in the study were in accordance with the ethical standards of the institutional and/or national research committee (Health Research Ethics Committee of the North-West University; NWU-00007-15-A1) and with the 1964 Helsinki Declaration and its later amendments or comparable ethical standards (Carlson et al. 2004).

### GC–MS conditions

All analyses were performed on a GC–MS apparatus consisting of a single quadrupole mass spectrometer model ISQ, a Trace 1210 series gas chromatograph and an AS1310 auto-sampler from ThermoFisher (Dreieich, Germany). A fused-silica capillary column Optima 17 (15 m length, 0.25 mm I.D., 0.25 µm film thickness) from Macherey–Nagel (Düren, Germany) was used. Toluene aliquots (1 µL) were injected in the split-less mode. The 10 µL Hamilton needle of the autosampler was cleaned automatically three times with toluene (5 µL) after each injection. Injector temperature was kept at 280 °C. Helium was used as the carrier gas at a constant flow rate of 1.0 mL/min. The oven temperature was held at 40 °C for 0.5 min and ramped to 210 °C at a rate of 15 °C/min and then to 320 °C at a rate of 35 °C/min. Interface and ion-source temperatures were set to 300 °C and 250 °C, respectively. Electron energy was 70 eV and electron current 50 µA. Methane was used as the reagent gas for negative-ion chemical ionization (NICI) at a constant flow rate of 2.4 mL/min. Mass spectra of amino acid derivatives were generated by scanning the quadrupole in the *m*/*z *range 50–1000. In quantitative analyses, the dwell time was 50 ms or 100 ms for each ion in the selected-ion monitoring (SIM) mode and the electron multiplier voltage was set to 1400 V. The mass spectra of the amino acid derivatives and the ions monitored in quantitative analyses are reported in the “Results” section and in the Supplement.

### Statistical analyses

Data analyses were performed using SPSS version 21 (SPSS Inc., Chicago, IL, USA) and GraphPad Prism 7 for Windows (GraphPad Software, San Diego, CA, USA). Continuous variables are presented as mean ± SD or median with interquartile range (IQR) as appropriate. Categorical variables are reported as proportions (*n*, %). Comparison between groups was performed using unpaired *t* test, Mann–Whitney *U* test or Fisher’s exact test as appropriate. Pearson or Spearman’s correlation analyses were used to test for associations between plasma and urine amino acids. Within all statistical analyses, a two-sided *P* value of less than 0.05 was considered statistically significant. GraphPad Prism 7 was used to prepare graphs and receiver operating characteristic (ROC) curves.

## Results

### GC–MS characterization of the derivatives of Lys and its PTM metabolites of methylation and hydroxylation

Derivatization of amino acids (AA) and their metabolites first with 2 M HCl/CH_3_OH and then with PFPA/EA yields the methyl ester (Me) *N*-pentafluoropropionyl (PFP) derivatives (d_0_Me)_*m*_-AA-(PFP)_*n*_, where *m* is the number of esterified carboxyl groups and *n* is the number of the PFP-acylated amine groups. The general formula for the amino acids derivatives prepared with 2 M HCl/CD_3_OD and subsequently with PFPA/EA is (d_3_Me)_*m*_-AA-(PFP)_*n*_. GC–MS spectra were generated from separately derivatized and analysed amino acids and their metabolites. The same GC–MS conditions were used including the oven temperature program. The structures of the derivatives were elucidated on the basis of their mass spectra, the expected 3-Da difference between d_0_Me and d_3_Me per each carboxylic group in corresponding ions, and the expected shorter retention time (*t*_R_) of the deuterium-containing derivatives. As Lys was of particular interest in our study, the results from Lys and its metabolites are separately summarized in Table 1.

**Table 1:** GC–MS characteristics of the methyl ester (Me) pentafluoropropionyl (PFP) derivatives of l-lysine (K), its metabolites and their respective internal standards analyzed in the present study (*m*/*z* range 100–600)

Lysine/lysine metabolite	CH_3_OH or CD_3_OD	*t*_R_ (min)	Formula	Mass fragments (*m*/*z*) and their intensity (%) in GC–MS spectra
l-Lysine	CH_3_OH	9.48	d_0_Me-K-(PFP)_2_	**432 (100)**, 412 (40), 392 (10), 380 (6), 372 (5), 289 (15), 215 (5), 201 (45)
	CD_3_OD	9.46	d_3_Me-K-(PFP)_2_	**435 (100)**, 415 (25), 395 (10), 380 (6), 372 (3), 292 (15), 218 (5), 201 (40)
*N*^ε^-Methyl-l-lysine	CH_3_OH	9.77	d_0_Me-N^ε^MK-(PFP)_2_	**446 (100)**, 426 (6), 406 (5), 233 (12)
	CD_3_OD	9.75	d_3_Me-N^ε^MK-(PFP)_2_	**449 (100)**, 429 (6), 409 (3), 236 (10)
*N*^α^-Methyl-l-lysine	CH_3_OH	10.35	d_0_Me-N^α^MK-(PFP)_2_	426, 406, 366, 289, 238, 220, **188 (100**)
	CD_3_OD	10.34	d_3_Me-N^α^MK-(PFP)_2_	429, 409, 369, 292, 241, 223, **188 (100)**
*N*^ε^-Acetyl-l-lysine	CH_3_OH	11.84	d_0_Me-N^ε^AcK-PFP	**328 (100)**, 308 (17), 288 (18), 270 (55), 249 (12), 231 (17)
	CD_3_OD	11.82	d_3_Me-N^ε^AcK-PFP	**331 (100)**, 311 (12), 291 (20), 273 (70), 253 (12), 231 (10)
*N*^α^-Acetyl-l-lysine	CH_3_OH	11.95	d_0_Me-N^α^AcK-PFP	**328 (100)**, 288 (35), 289 (30), 269 (30)
	CD_3_OD	11.93	d_3_Me-N^α^AcK-PFP	**331 (100)**, 292 (25), 272 (40)
*N*^ε^,*N*^ε^-Dimethyl-l-lysine	CH_3_OH	8.08	d_0_Me-N^ε^N^ε^MK-PFP	314 (7), **188 (100)**
	CD_3_OD	8.07	d_3_Me-N^ε^N^ε^MK-PFP	317 (5), **188 (100)**
*N*^ε^,*N*^ε^,*N*^ε^-Trimethyl-l-lysine	CH_3_OH	no peaks		
	CD_3_OD			
D,l-5-Hydroxy-l-lysine	CH_3_OH	8.55	d_0_Me-5OHK-(PFP)_3_	594 (18), 448 (42), 430 (5), 233 (10), 214 (5), **163 (100)**, 128 (11)
	CD_3_OD	8.53	d_3_Me-5OHK-(PFP)_3_	597 (15), 451 (50), 433 (5), 236 (15), 214 (4), **163 (100)**, 128 (11)
D,l-5-Hydroxy-l-lysine	CH_3_OH	8.66	d_0_Me-5OHK-(PFP)_3_	594 (22), 448 (60), 430 (5), 233 (20), 214 (5), **163 (100)**, 128 (18)
	CD_3_OD	8.64	d_3_Me-5OHK-(PFP)_3_	597 (20), 451 (60), 433 (5), 236 (16), 214 (4), **163 (100)**, 128 (12)

Bold values indicate the most intense ions

Due to its single carboxylic group and the two amine groups, the derivatization of unlabelled and labelled Lys (K) yielded (d_0_Me)_1_-K-(PFP)_2_ and (d_3_Me)_1_-K-(PFP)_2_. The derivatives eluted at 9.48 min and 9.46 min, and their calculated molecular masses (M) are 452 and 455, respectively. The most intense ions in their GC–MS spectra were *m*/*z* 432 [M-HF]^−^ and *m*/*z* 435 [M-HF]^−^ due to neutral loss each of a HF (20 Da) group from the molecular anions [M]^−^ at *m*/*z* 452 at *m*/*z* 455, respectively (see supplementary Fig. S1). The GC–MS spectra of the derivatives contained further mass fragments which differed by 3 Da and other mass fragments which did not differ in their *m*/*z* values (Table 1).

**Fig. 2:**
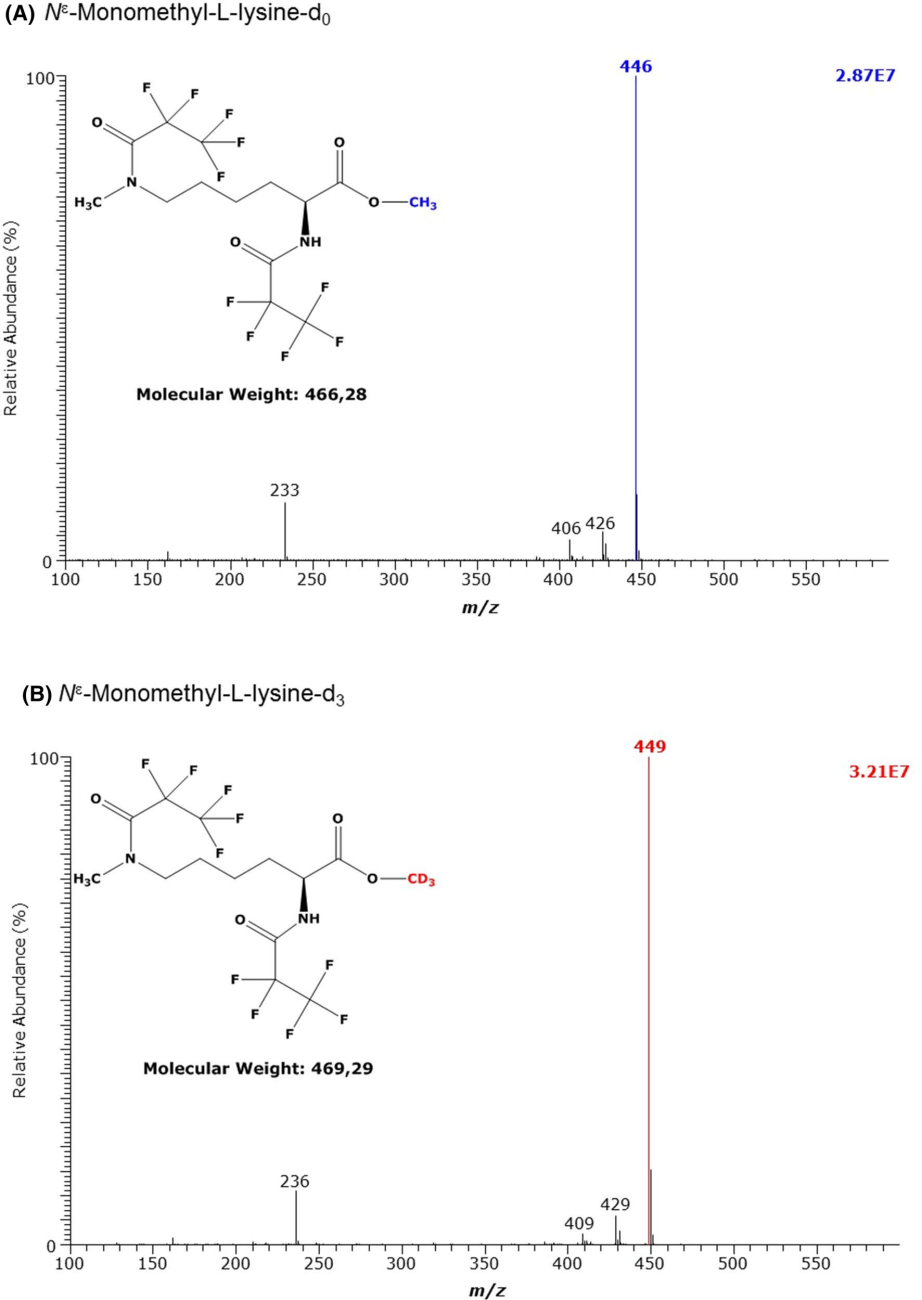
GC–MS spectra of the methyl ester pentafluoropropionyl derivates of **A** the unlabelled and **B** the deuterium-labelled *N*^ε^-monomethyl-l-lysine (MML)

**Fig. 3:**
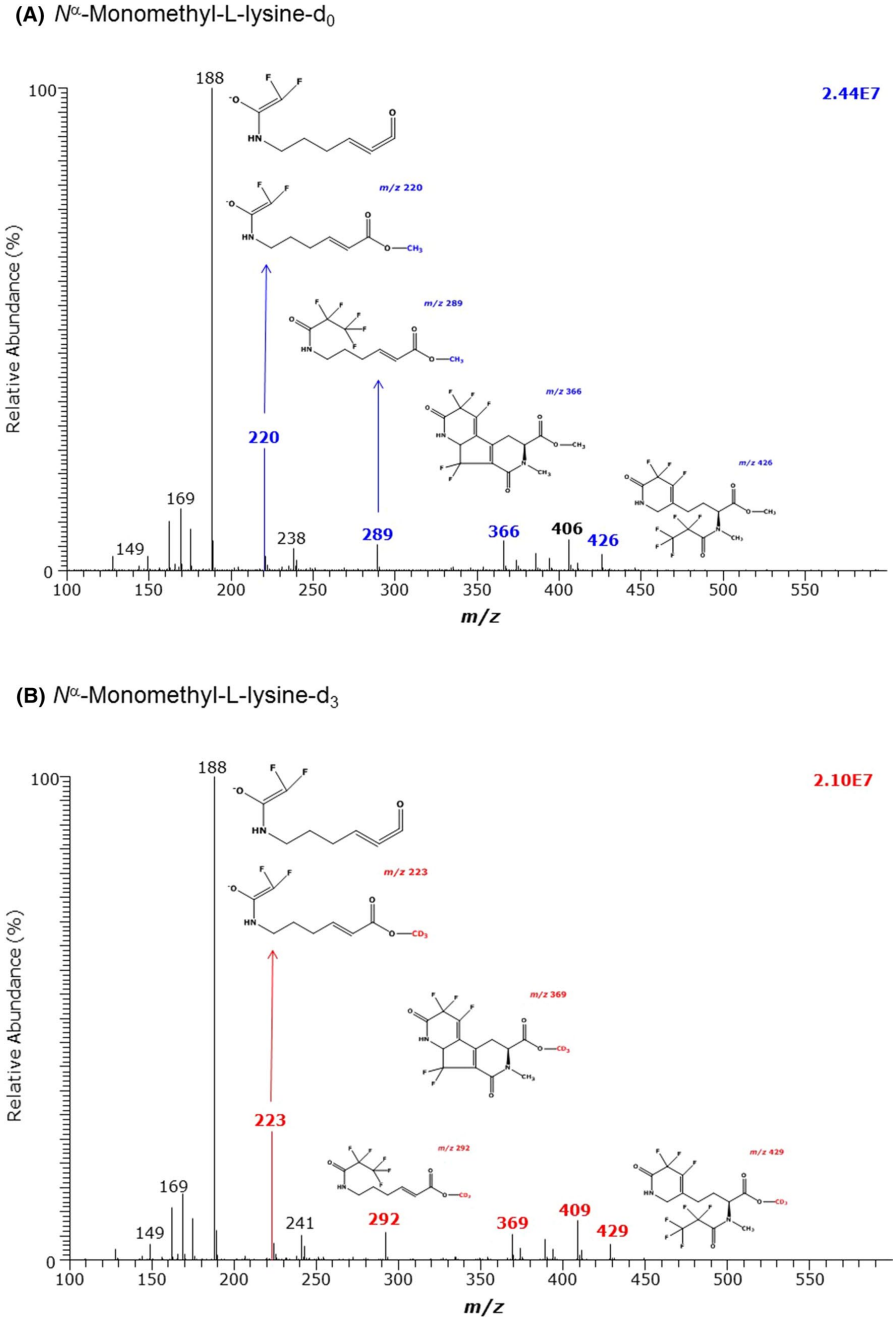
GC–MS spectra of the methyl ester pentafluoropropionyl derivates of **A** the unlabelled and **B** the deuterium-labelled *N*^α^-methyl-l-lysine (MML)

The derivatization of synthetic *N*^ε^-methyl-l-lysine Lys, i.e., N^ε^MK, yielded (d_0_Me)_1_-N^ε^MK-(PFP)_2_ (M, 466) and (d_3_Me)_1_-N^ε^MK-(PFP)_2_ (M, 469) which eluted at 9.77 min and 9.75 min, respectively, i.e., behind the derivatives of Lys. The most intense anions were *m*/*z* 446 and *m*/*z* 449 ([M-HF]^−^), respectively (Table 1), whereas no molecular anions [M]^−^ were present in both mass spectra (Fig. 2). The derivatization of synthetic *N*^α^-methyl-l-lysine Lys, i.e., *N*^α^MK, yielded (d_0_Me)_1_-N^α^MK-(PFP)_2_ (M, 466) and (d_3_Me)_1_-N^α^MK-(PFP)_2_ (M, 469) which eluted at 10.35 min and 10.34 min, respectively, i.e., considerably later than the derivative of the isomeric *N*^ε^-methyl-l-lysine. The GC–MS spectra of (d_0_Me)_1_-N^α^MK-(PFP)_2_ and (d_3_Me)_1_-N^α^ML-(PFP)_2_ contained the most intense ions at *m*/*z* 188 and less intense mass fragments differing by 3 Da, with *m*/*z* 220 and *m*/*z* 223 being the most intense (Fig. 3, Table 1). Thus, Fig. 2 and Fig. 3 indicate that the isomeric *N*-methyl-lysine metabolites N^ε^MK and N^α^MK can be discriminated by GC–MS when analyzed as their methyl ester pentafluoropropionyl derivatives.

**Fig. 4:**
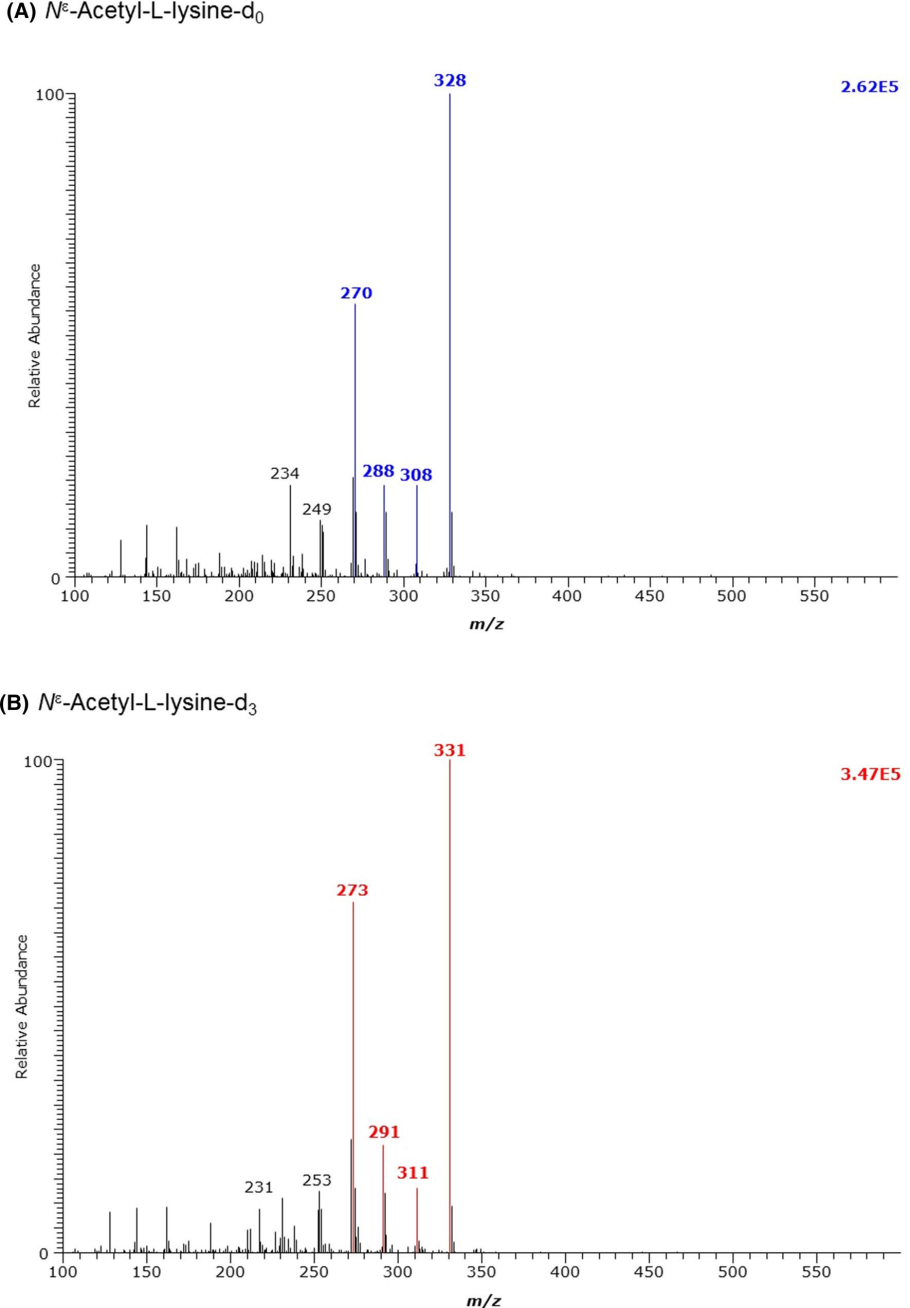
GC–MS spectra of the methyl ester pentafluoropropionyl derivates of **A** the unlabelled and **B** the deuterium-labelled *N*^ε^-acetyl-l-lysine (N^ε^AcL)

The derivatization of *N*^ε^-acetyl-l-lysine (*N*^ε^AcK) and *N*^α^-acetyl-l-lysine (*N*^α^AcK) under the same conditions was associated with abundant decomposition, with the main derivatives being those of lysine i.e., (d_0_Me)_1_-K-(PFP)_2_) (not shown). The mass spectrum of the GC–MS peak of *N*^ε^-acetyl-l-lysine (N^ε^AcK) was about 100 times less intense than that of the lysine derivative. The mass spectrum of the GC–MS peak of *N*^α^-acetyl-l-lysine (N^α^AcK) was even about 1000 times less intense than that of the lysine derivative (Fig. 4, Table 1). These results indicate that *N*^ε^-acetyl-l-lysine is considerably more stable than the *N*^α^-acetyl-l-lysine. Because the derivatives are separated chromatographically, *N*^ε^-acetyl-l-lysine could be better quantifiable in biological samples than *N*^α^-acetyl-l-lysine.

**Fig. 5:**
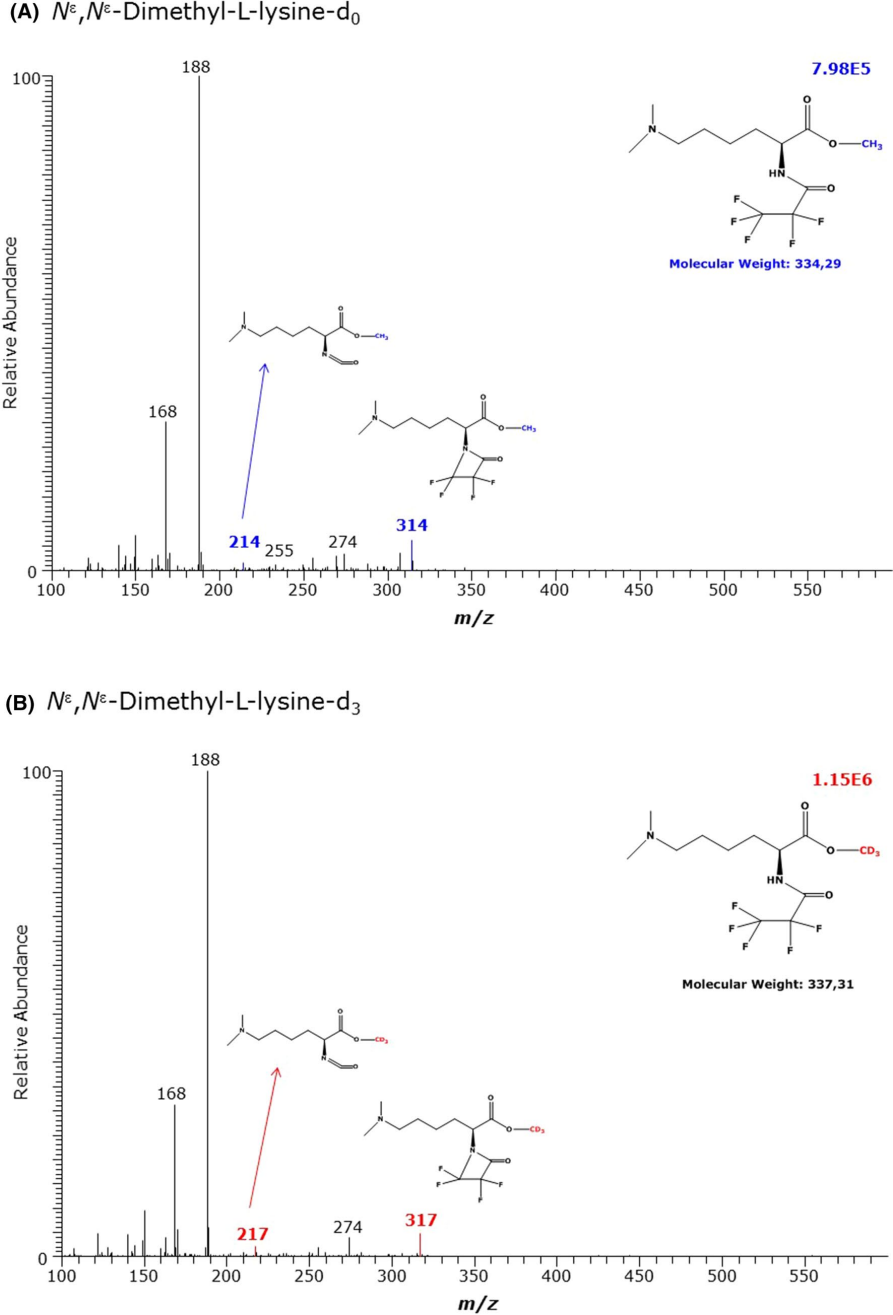
GC–MS spectra of the methyl ester pentafluoropropionyl derivates of **A** the unlabelled and **B** the deuterium-labelled *N*^ε^,*N*^ε^-dimethyl-l-lysine (DML)

The derivatization of *N*^ε^,*N*^ε^-dimethyl-l-lysine (N^ε^N^ε^MK) resulted in very small peaks with retention times of about 8.08 min of which the GC–MS spectra are shown in Fig. 5 and Table 1. The most intense mass fragments *m*/*z* 188 and *m*/*z* 168 were common to the non-deuterated and deuterated methyl esters and are not specific for the *N*^ε^*N*^ε^MK derivatives as they were also observed in the GC–MS spectra of the *N*^α^-methyl-l-lysine derivatives. The GC–MS spectra of d_0_Me-N^ε^N^ε^MK-PFP and d_3_Me-N^ε^N^ε^MK-PFP contain less intense mass fragments which differ by 3 Da, i.e., *m*/*z* 314 and *m*/*z* 317, and *m*/*z* 214 and *m*/*z* 217, and are therefore suitable for quantitative analysis of *N*^ε^,*N*^ε^-dimethyl-l-lysine (Fig. 5). *N*^ε^*-*Methyl-l-lysine and *N*^ε^,*N*^ε^-dimethyl-l-lysine were found not to decompose to l-lysine. The derivatization of synthetic *N*^ε^,*N*^ε^,*N*^ε^*-*trimethyl-l-lysine did not result in any derivative extractable into toluene. This observation suggests that *N*^ε^,*N*^ε^,*N*^ε^*-*trimethyl group is stable under the derivatization conditions and, because of the permanent positive charge of *N*^ε^,*N*^ε^,*N*^ε^*-*trimethyl-l-lysine, even formation of d_0_Me-N^ε^N^ε^N^ε^MK-PFP and d_3_Me-N^ε^N^ε^N^ε^MK-PFP will not allow extraction of these derivatives into toluene or any other water-immiscible organic solvents compatible with GC–MS.

5-Hydroxy-l-lysine is an enzymatic PTM metabolite of Lys residues in proteins. The most common 5-hydroxylysine stereoisomer found in collagen is 5(*R*)-hydroxy-l-lysine (Gjaltema and Bank 2017). The Me-PFP derivatives of synthetic 5(S,*R*)-hydroxy-l-lysine eluted as almost baseline-separated peaks of comparable size at 8.55 min and 8.66 min for unlabelled and 8.53 min and 8.64 min for the deuterium-labelled derivatives and had virtually identical GC–MS spectra (Fig. S2, Table 1). Several ions differed by 3 Da thus allowing quantitative analyses of both isomers. The GC–MS results strongly suggest that 5-hydroxy-lysine is converted to its methyl ester tris-pentafluoropropionyl derivatives d_0_Me-5-hydroxy-Lys-(PFP)_3_ and d_3_Me-5-hydroxy-Lys-(PFP)_3_. As Me-PFP derivatives of racemic amino acids are chromatographically not separated in our system (Hanff et al. 2019), the elution of the racemic d_0_Me-5-hydroxy-Lys-(PFP)_3_ and d_3_Me-5-hydroxy-Lys-(PFP)_3_ as double almost baseline-separated peaks suggests that the separation is due to the derivatized 5-hydroxy group of 5-hydroxy-l-lysine.

### GC–MS characterization of the derivatives of l-Lys-derived AGEs

**Fig. 6:**
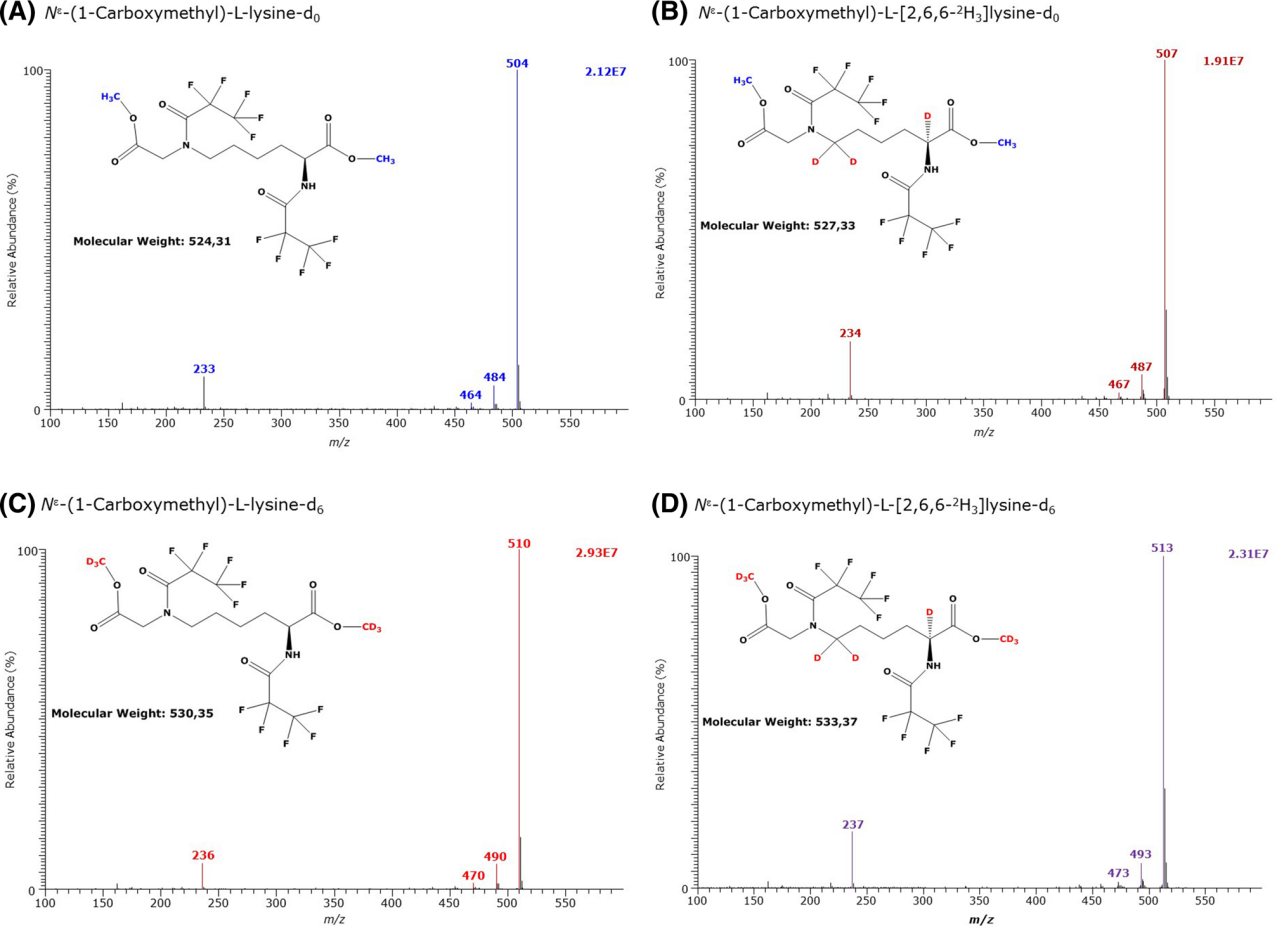
GC–MS spectra of the methyl ester pentafluoropropionyl derivates of **A** the unlabelled *N*^ε^-(1-carboxymethyl)-l-lysine (CML), **B** the *N*^ε^-(1-carboxymethyl)-l-[2,6,6,-^2^H_3_]lysine, and of the deuterium-labelled, **C** *N*^ε^-(1-carboxymethyl)-l-lysine and **D** *N*^ε^-(1-carboxymethyl)-l-[2,6,6,-^2^H_3_]lysine

**Fig. 7:**
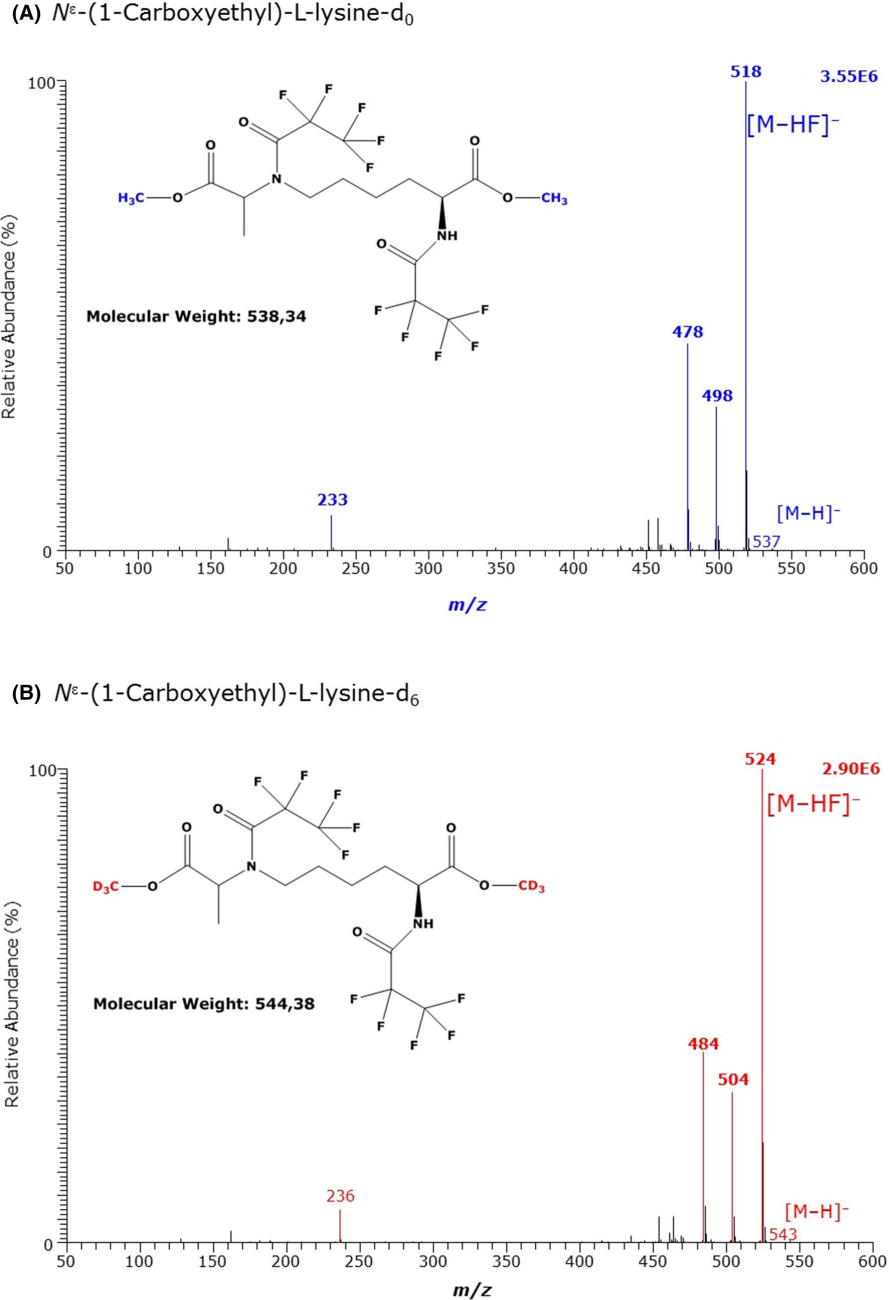
GC–MS spectra of the methyl ester pentafluoropropionyl derivates of **A** the unlabelled and **B** the deuterium-labelled *N*^ε^-(1-carboxyethyl)-l-lysine (CEL)

**Fig. 8:**
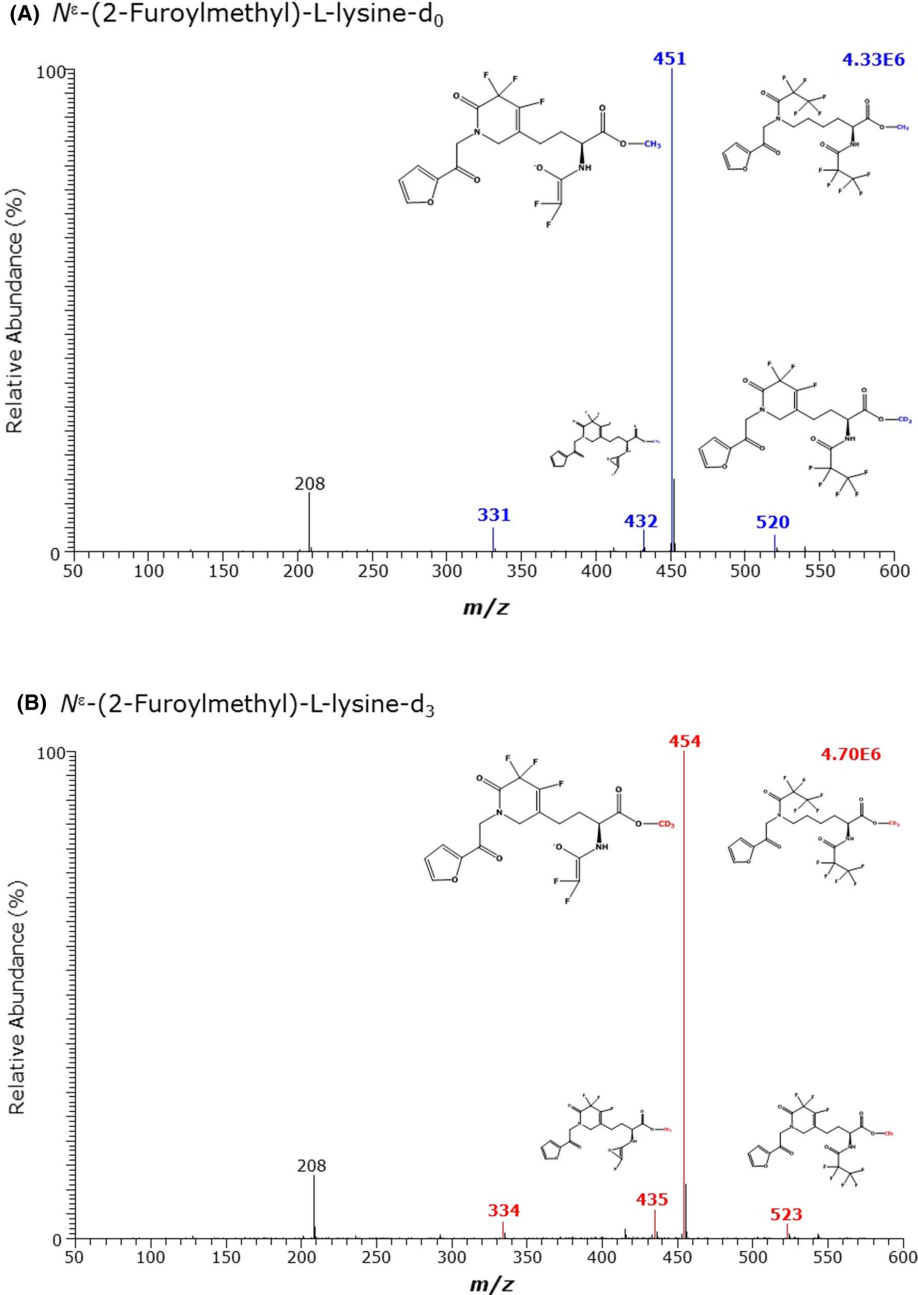
GC–MS spectra of the methyl ester pentafluoropropionyl derivates of **A** the unlabelled and **B** the deuterium-labelled *N*^ε^-(2-furoylmethyl)-l-lysine (furosine)

**Fig. 9:**
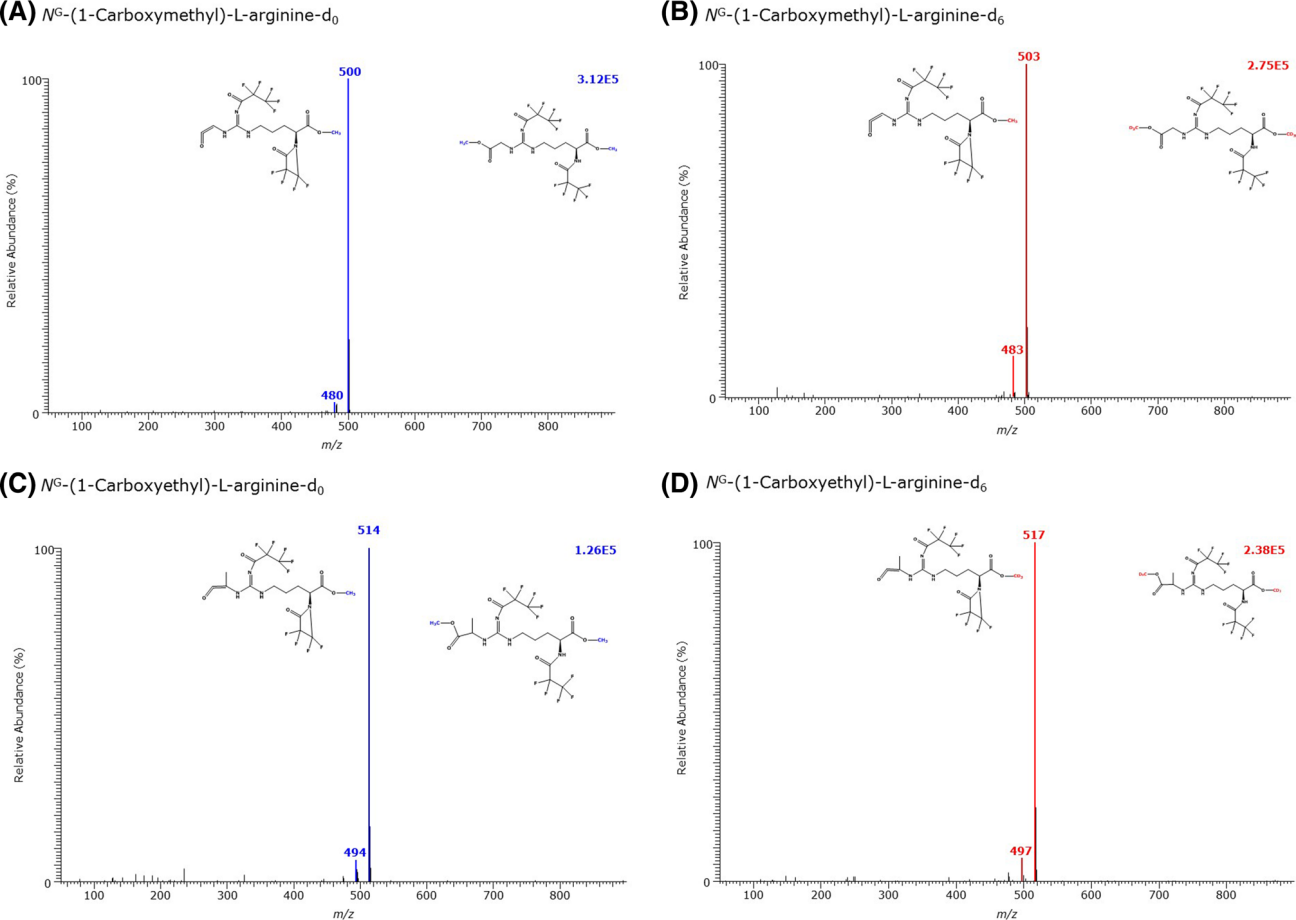
GC–MS spectra of the methyl ester pentafluoropropionyl derivates of **A** the unlabelled and **B** the deuterium-labelled *N*^G^-(1-carboxymethyl)-l-arginine (CMA), and **C** of the unlabelled and **D** the deuterium-labelled *N*^G^-(1-carboxyethyl)-l-arginine (CEA)

**Fig. 10:**
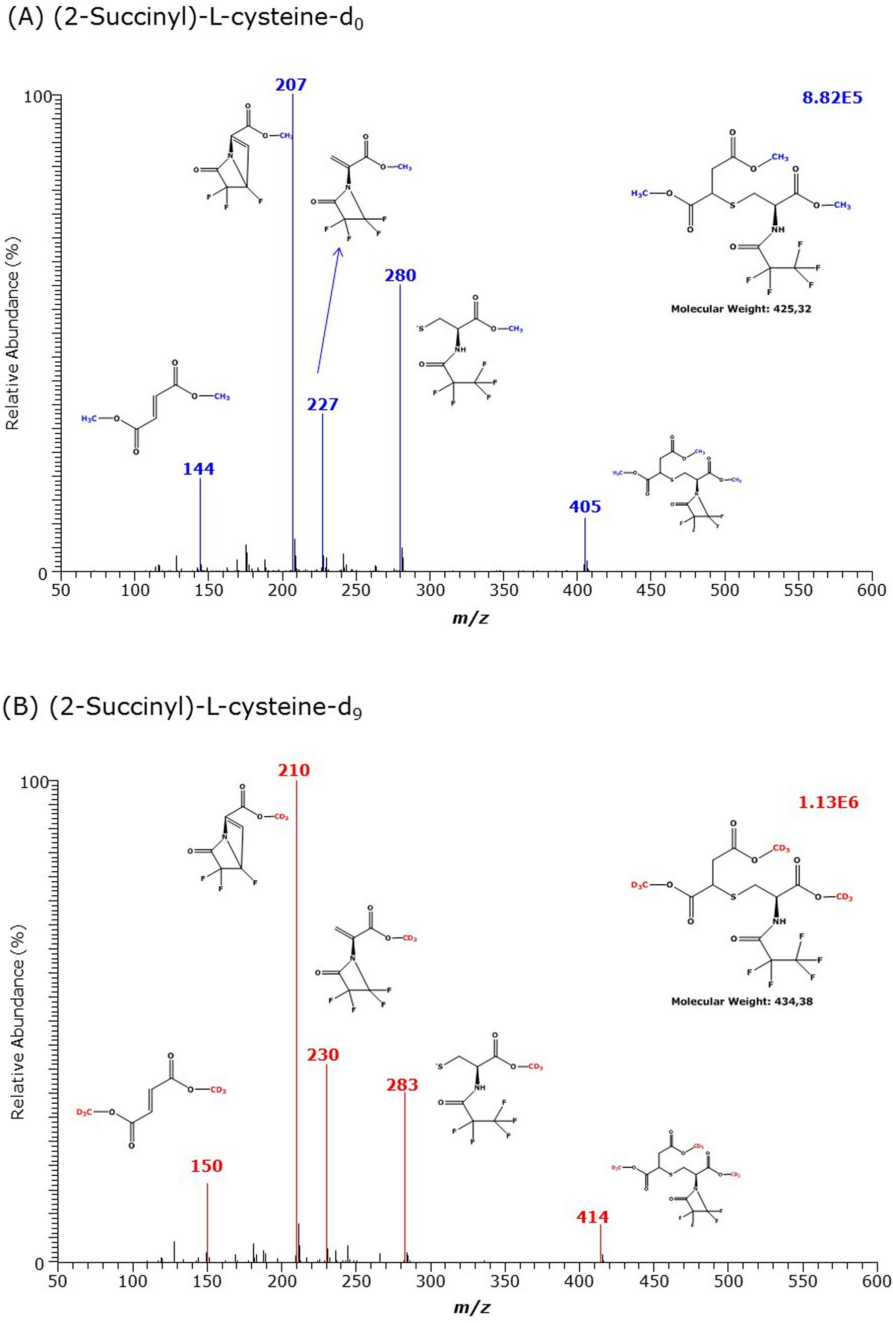
GC–MS spectra of the methyl ester pentafluoropropionyl derivates of unlabelled (upper panel) and deuterium-labelled (lower panel) of (2-succinyl)-l-cysteine (S2C). Insets indicate the proposed structures of the derivates and the mass fragments

As AGEs derived from Lys, Arg and Cys are carboxylic acids and contain amine groups, we tested the utility of GC–MS for their quantitative analysis as Me-PFP derivatives using in situ prepared or commercially available stable-isotope labelled analogs. The results are summarized in Table 2 and in Figs. 6, 7, 8, 9 and 10.

**Table 2:** GC–MS characteristics of the methyl ester (Me) pentafluoropropionyl (PFP) derivatives of the AGEs of l-lysine (K), l-arginine (R) and l-cysteine (C), and of their respective internal standards analyzed in the present study

AGE	CH_3_OH or CD_3_OD	*t*_R_ (min)	Formula	Mass fragments (*m*/*z*) and their intensity (%) in the GC–MS spectra
*N*^ε^-(1-Carboxymethyl)-l-lysine	CH_3_OH	11.46	(d_0_Me)_2_-CMK-(PFP)_2_	**504 (100)**, 484 (8), 464 (2), 233 (10)
	CD_3_OD	11.42	(d_3_Me)_2_-CEK-(PFP)_2_	**510 (100)**, 490 (7), 470 (2), 236 (7)
*N*^ε^-(1-Carboxymethyl)-l-[2,4,4-^2^H_3_]-lysine	CH_3_OH	11.44	(d_0_Me)_2_-d_3_CMK-(PFP)_2_	**507 (100)**, 487 (7), 467 (2), 234 (15)
	CD_3_OD	11.40	(d_3_Me)_2_-d_3_CMK-(PFP)_2_	**513 (100)**, 493 (7), 473 (2), 237 (15)
*N*^ε^-(1-Carboxyethyl)-l-lysine	CH_3_OH	11.37	(d_0_Me)_2_-CEK-(PFP)_2_	**518 (100)**, 498 (35), 478 (45), 233 (8)
	CD_3_OD	11.33	(d_3_Me)_2_-CEK-(PFP)_2_	**524 (100)**, 504 (35), 484 (42), 236 (7)
*N*^ε^-(2-Furoylmethyl)-l-lysine	CH_3_OH	13.39	(d_0_Me)_2_-FMK-(PFP)_2_	520 (4), **451 (100)**, 432 (5), 331 (5), 208 (12)
	CD_3_OD	13.38	(d_3_Me)_2_-FMK-(PFP)_2_	523 (4), **454 (100**), 435 (5), 334 (4), 208 (14)
*N*^G^-(1-Carboxymethyl)-l-arginine	CH_3_OH	13.62	(d_0_Me)_2_-CMR-(PFP)_2_	**500 (100)**, 480 (4)
	CD_3_OD	13.61	(d_3_Me)_2_-CER-(PFP)_2_	**503 (100)**, 483 (12)
*N*^G^-(1-Carboxyethyl)-l-arginine	CH_3_OH	13.11	(d_0_Me)_2_-CER-(PFP)_2_	**514 (100)**, 494 (7)
	CD_3_OD	13.11	(d_3_Me)_2_-CER-(PFP)_2_	**517 (100)**, 497 (7)
(*S*-Carboxymethyl)-l-cysteine	CH_3_OH	10.00	(d_0_Me)_2_-CMC-(PFP)_1_	333 (2), 313 (2), 280 (20), 227 (35), **207 (100),** 104
	CD_3_OD	9.96	(d_3_Me)_2_-CMC-(PFP)_1_	339 (2), 319 (2), 283 (20), 230 (40), **210 (100),** 107
(*S*-Carboxyethyl)-l-cysteine	CH_3_OH	11.90	(d_0_Me)_2_-CEC-(PFP)_1_	278 (100), 128 (85)
	CD_3_OD	11.86	(d_3_Me)_2_-CEC-(PFP)_1_	281 (100), 128 (60)
(2-Succinyl)-l-cysteine	CH_3_OH	12.18	(d_0_Me)_3_-SC-(PFP)_1_	405 (12), 280 (62), 227 (32), **207 (100)**, 175 (6), 144 (20)
	CD_3_OD	12.13	(d_3_Me)_3_-SC-(PFP)_1_	414 (7), 283 (36), 230 (42), **210 (100)**, 181 (5), 150 (16)

Bold values indicate the most intense ions

*CM* carboxymethyl, *CE* carboxyethyl, *FM* 2-furoylmethyl, *SC* (2-succinyl)-l-cysteine, *t*_*R*_ retention time

GC–MS analysis of the unlabelled and deuterium-labelled *N*^ε^-(1-carboxymethyl)-l-lysine (N^ε^CMK) derivatives resulted in the formation of closely eluting GC–MS peaks around 11.4 min without evidence of decomposition to Lys. The mass spectra of these GC–MS peaks are shown in Fig. 6 and Table 2 and can be reliably assigned to the dimethyl esters–dipentafluoropropionyl derivatives: (d_0_Me)_2_-N^ε^CMK-(PFP)_2_ (M, 524) and (d_3_Me)_2_-N^ε^CMK-(PFP)_2_ (M, 530), (d_0_Me)_2_-N^ε^Cd_3_MK-(PFP)_2_ (M, 527) and (d_3_Me)_2_-N^ε^Cd_3_MK-(PFP)_2_ (M, 533).

GC–MS analysis of unlabelled and deuterium-labelled *N*^ε^-(1-carboxyethyl)-l-lysine (N^ε^CEK) resulted in the formation of the derivatives of l-lysine, suggesting decomposition of N^ε^CEK at its *N*^ε^ amine group to form the Lys derivative. The most intense GC–MS peaks from N^ε^CEK derivatization were observed at 11.37 min and 11.33 min (Fig. 7, Table 2). These GC–MS spectra suggest formation of dimethyl ester-dipentafluoropropionyl derivatives of N^ε^CEK, i.e., (d_0_Me)_2_-N^ε^CEK-(PFP)_2_ (M, 538) and (d_3_Me)_2_-N^ε^CEK-(PFP)_2_ (M, 544). These results suggest that the methyl group of N^ε^CEK on its terminal carboxylic greatly contributes to its instability during the derivatization processes.

*N*^ε^-(2-Furoylmethyl)-l-lysine (N^ε^FMK, furosine) is the AGE of l-lysine with fructose. Derivatization of synthetic N^ε^FMK resulted in the formation of a single GC–MS peak eluting at about 13.4 min. This is the longest retention time of Lys and its metabolites (Tables 1, 2). There was no formation of the Lys derivative during the derivatization steps suggesting a stable N^ε^FMK derivative at the *N*^ε^-(2-furoylmethyl) residue (data not shown). The most intense mass fragments were *m*/*z* 451 for the unlabelled N^ε^FMK and *m*/*z* 454 for the deuterium-labelled N^ε^FMK (Fig. 8, Table 2).

### GC–MS characterization of the derivatives of Arg- and Cys-derived AGEs

Under the same derivatization procedures and GC–MS conditions, the derivatives of synthetic *N*^G^-(1-carboxymethyl)-l-arginine and *N*^G^-(1-carboxyethyl)-l-arginine eluted as very small GC–MS peaks that contained each a single intense mass fragment (Fig. 9, Table 2). Interestingly, the carboxyethyl derivatives of Arg and Lys eluted in front of the corresponding carboxymethyl derivatives. No derivatives of Arg were observed from derivatized *N*^G^-(1-carboxymethyl)-l-arginine and *N*^G^-(1-carboxyethyl)-l-arginine suggesting a higher stability of the *N*^G^-(1-carboxymethyl)-l-arginine and *N*^G^-(1-carboxyethyl)-l-arginine derivatives compared to the corresponding Lys derivatives.

*S*-(2-Succinyl)-l-cysteine (SC) derivatization resulted in elution each of a relatively large GC–MS peak at 12.18 min (unlabelled) and 12.13 min (labelled) of which the spectra contained mass fragments differing by 3 Da, 6 Da and 9 Da (Fig. 10). These results suggest formation of (d_0_Me)_3_-SC-(PFP)_1_ and (d_3_Me)_3_-SC-(PFP)_1_, respectively. Derivatization of the commercially available (*S*-carboxymethyl)-l-cysteine (CMC) yielded a small GC–MS peak eluting at about 10 min (Fig. S3). A very small GC–MS peak was obtained from derivatized (*S*-carboxyethyl)-l-cysteine (CEC) eluting at 11.8 min (Fig. S4, Table 2). These results indicate decomposition during the derivatization steps.

### Method validation in human urine

The GC–MS method was validated using 10-µL aliquots of a pooled human urine sample in relevant concentration ranges for all analytes on three consecutive days in triplicate for each concentration. After sampling for the first validation day, the pooled urine sample was frozen at − 20 °C. This procedure was repeated on the next two days. The respective internal standards were in situ prepared using a mixture of the unlabelled analytes and derivatization in 2 M HCl/CD_3_OD to finally achieve concentrations being within the respective ranges of the analytes. Of the final toluene extracts, 1-µL aliquots were injected split-less and selected-ion monitoring (SIM) was performed. The same dwell-time was used for each analyte and its internal standard. The peak areas of analytes and internal standard were used for quantification. The concentration of each analyte was calculated by multiplying the PAR of analyte-to-internal standard and by the known concentration of the respective internal standard. The precision of the method was expressed as the relative standard deviation (RSD, %) from the triplicate analyses. The accuracy of the method was determined for the added analyte concentrations by subtracting the respective endogenous (basal) concentration in the un-spiked urine sample, dividing the difference with the respective added concentration and multiplying the outcome by 100. The accuracy of the method was expressed as recovery (%). Linear regression analysis was performed between measured analyte concentration (*y*) and added analyte concentration (*x*). The *y-*axis intercept of the regression equation provides the mean analyte concentration in the urine sample used in method validation. The slope value of the regression equation multiplied by 100 yields the mean recovery value for the analyte in the investigated concentration range. In total, the concentrations of 33 amino acids and their metabolites were determined simultaneously. Table 3 lists the SIM pairs used in quantitative analyses including the method validation.

**Table 3:** Summary of the GC–MS conditions used for the simultaneous quantitative determination of the indicated amino acids and their metabolites (AA) in human urine using their stable-isotope labelled analogs as their internal standards (IS)

Amino acid (AA)	AA/IS (*m*/*z*)	Retention time (min)	Dwell time (ms)	Time window (min)
Alanine	229/232	3.73/3.70	100	3.60–3.90
Threonine	259/262	4.07/4.05	50	3.90–4.80
Glycine	215/218	4.22/4.20	50	
Valine	257/260	4.44/4.42	50	
Serine	207/210	4.46/4.43	50	
Sarcosine	229/232	4.98 /4.96	100	4.80–5.40
Leucine/isoleucine	271/274	5.09/5.07	100	
Guanidininoacetate	383/386	6.67/6.65	50	6.40–7.05
Asparagine/aspartate	287/293	6.74/6.69	50	
4-Hydroxy-proline	397/400	6.83/6.81	50	
Proline	255/258	7.18/7.16	100	7.05–7.50
Glutamine/glutamate	301/307	7.93/7.89	100	7.50–8.30
Methionine	289/292	7.94/7.92	100	
5-Hydroxy-lysine (1st peak)	448/451	8.50/8.49	100	8.30–8.85
Ornithine/citrulline	418/421	8.60/8.58	50	8.30–8.85
5-Hydroxy-lysine (2nd peak)	448/451	8.61/8.59	50	
Phenylalanine	305/308	8.73/8.71	50	
Tyrosine	233/236	9.06/9.04	100	8.85–9.30
Lysine	432/425	9.51/9.49	50	9.30–9.90
Arginine	586/589	9.60/9.58	50	
*N*^ε^-Monomethyl-lysine	446/449	9.81/9.79	50	
*S*-(2-Carboxymethyl)-cysteine	104/107	10.04/10.00	100	9.90–10.80
Homoarginine	600/603	10.39/10.37	100	
*N*^ε^-(2-Carboxyethyl)-lysine	518/524	11.40/11.36	50	10.80–12.00
Tryptophan	233/236	11.48/11.45	50	
*N*^ε^-(2-Carboxymethyl)-lysine	504/510	11.49/11.45	50	
*N*^G^,*N′*^G^-Dimethyl-arginine	634/637	11.56/11.54	50	
*S*-(2-Carboxyethyl)-cysteine	278/281	11.90/11.86	50	
*S*-(2-Succinyl)-cysteine	405/414	12.24 /12.19	100	12.00–12.40
*N*^G^-Carboxyethyl-arginine	514/517	13.13/13.12	50	12.85–13.90
*N*^G^-Monomethyl-arginine	474/477	13.24/13.23	50	
*N*^ε^-(2-Furoylmethyl)-lysine	451/454	13.42 /13.41	50	
*N*^G^-Carboxyethyl-arginine	500 /503	13.63/13.62	50	

The results from the method validation for all analytes are listed in Table S1 in the Supplement to this article. For the sake of simplicity and clarity, the results of the validation method for Lys, Arg, Cys and their PTM metabolites and AGEs are presented in Table 4.

**Table 4:** Summary of the results of the intra- and inter-day validation (precision, RSD) and accuracy (recovery) of the GC–MS method for the simultaneous measurement of l-lysine, l-arginine, l-cysteine, and their PTM and AGE metabolites in human urine in the indicated biologically relevant concentration ranges using their deuterium-labelled methyl esters analogs as internal standards

Amino acid Amino acid PTM Amino acid AGE	Range (µM)	Day 1			Day 2			Day 3		
	Added (µM)	Measured (µM)	RSD (%)	Recovery (%)	Measured (µM)	RSD (%)	Recovery (%)	Measured (µM)	RSD (%)	Recovery (%)
l-Lysine	0–275	*y* = 0.989*x* + 66.7, *r*^2^ = 0.999			*y* = 0.963*x* + 66.2, *r*^2^ = 0.9983			*y* = 1.014*x* + 70.1, *r*^2^ = 0.9998		
*m*/*z* 432 *m*/*z* 435 (IS, 100 µM)	0	65.1 ± 2.93	4.50	n.a	67.1 ± 5.26	7.85	n.a	69.8 ± 1.04	1.49	n.a
	55	120 ± 3.43	2.85	100	116 ± 4.81	4.16	88.3	125 ± 0.91	0.73	100
	110	179 ± 1.38	0.77	103	172 ± 2.32	1.34	95.8	183 ± 1.93	1.06	103
	165	229 ± 5.35	2.34	99.0	226 ± 2.11	0.93	96.6	238 ± 2.67	1.12	102
	220	289 ± 0.96	0.33	102	284 ± 8.33	2.93	98.8	296 ± 6.27	2.12	103
	275	335 ± 5.8	1.73	98.0	326 ± 5.33	1.63	94.1	347 ± 4.6	1.33	101
*N*^*ε*^-Monomethyl-l-Lys	0–20	*y* = 0.968*x* + 1.77, *r*^2^ = 0.9998			*y* = 0.963*x* + 1.96, *r*^2^ = 1			*y* = 1.035*x* + 1.78, *r*^2^ = 0.9991		
*m*/*z* 446 *m*/*z* 449 (IS, 8 µM)	0	1.84 ± 0.06	3.29	n.a	1.96 ± 0.17	8.57	n.a	1.99 ± 0.033	1.64	n.a
	4	5.51 ± 0.03	0.60	92.0	5.82 ± 0.15	2.57	96.4	5.94 ± 0.087	1.47	98.6
	8	9.66 ± 0.08	0.84	97.8	9.64 ± 0.12	1.27	96.0	9.92 ± 0.08	0.80	99.1
	12	13.3 ± 0.29	2.23	95.1	13.5 ± 0.47	3.52	96.0	14 ± 0.19	1.34	99.7
	16	17.3 ± 0.17	0.96	96.5	17.5 ± 0.43	2.46	96.9	18.2 ± 0.2	1.07	101
	20	21.2 ± 0.26	1.21	96.7	21.2 ± 0.25	1.17	96.0	22.8 ± 0.23	1.01	104
*N*^*ε*^-(2-Carboxyethyl)-l-Lys	0–25	*y* = 1.124*x* + 5.38, *r*^2^ = 0.9989			*y* = 1.071*x* + 5.52, *r*^2^ = 0.9993			*y* = 1.136*x* + 5.75, *r*^2^ = 0.9988		
*m*/*z* 518 *m*/*z* 524 (IS, 10 µM)	0	6.11 ± 0.14	2.28	n.a	6.44 ± 0.72	11.2	n.a	6.61 ± 0.25	3.73	n.a
	5	11.3 ± 0.11	1.00	103	11.2 ± 0.43	3.79	96.1	11.9 ± 0.067	0.57	105
	10	17.1 ± 0.33	1.92	110	16 ± 0.39	2.47	95.4	16.9 ± 0.34	2.04	103
	15	21.9 ± 0.68	3.12	105	21.7 ± 0.5	2.33	101	22.7 ± 0.66	2.89	107
	20	28 ± 1.34	4.79	109	26.7 ± 0.9	3.37	101	29 ± 0.38	1.30	112
	25	33.3 ± 0.91	2.72	109	32.5 ± 1.19	3.65	104	33.8 ± 0.56	1.65	109
*N*^*ε*^-(2-Carboxymethyl)-l-Lys	0–25	*y* = 1.079*x* + 5.63, *r*^2^ = 0.999			*y* = 1.072*x* + 5.88, *r*^2^ = 0.9983			*y* = 1.154*x* + 5.95, *r*^2^ = 0.9989		
*m*/*z* 504 *m*/*z* 510 (IS, 10 µM)	0	6.26 ± 0.31	4.88	n.a	6.77 ± 0.49	7.19	n.a	7.13 ± 0.088	1.23	n.a
	5	11.2 ± 0.22	1.96	99.8	11.5 ± 0.56	4.93	93.8	12.2 ± 0.067	0.55	101
	10	16.8 ± 0.19	1.14	105	16.5 ± 0.25	1.53	97.2	17.1 ± 0.3	1.77	99.4
	15	21.9 ± 0.85	3.91	104	22.5 ± 0.94	4.19	105	22.9 ± 0.59	2.57	105
	20	27.2 ± 0.42	1.54	105	26.7 ± 0.85	3.17	100	29 ± 0.24	0.82	110
	25	32.4 ± 0.61	1.90	104	32.9 ± 0.3	0.90	104	35.1 ± 0.58	1.65	112
*N*^ε^-(2-Furoylmethyl)-l-Lys	0–15	*y* = 0.961*x* + 0.826, *r*^2^ = 0.9994			*y* = 0.989*x* + 0.783, *r*^2^ = 0.9992			*y* = 1.017*x* + 0.765, *r*^2^ = 0.9985		
*m*/*z* 451 *m*/*z* 454 (IS, 6 µM)	0	0.99 ± 0.05	4.89	n.a	1.01 ± 0.1	9.75	n.a	1.04 ± 0.014	1.34	n.a
	3	3.58 ± 0.04	1.08	86.2	3.63 ± 0.1	2.65	87.4	3.72 ± 0.033	0.90	89.4
	6	6.56 ± 0.08	1.21	92.8	6.5 ± 0.1	1.48	91.4	6.54 ± 0.16	2.46	91.6
	9	9.42 ± 0.15	1.58	93.6	9.63 ± 0.2	2.03	95.8	9.82 ± 0.23	2.35	97.5
	12	12.2 ± 0.08	0.61	93.8	12.7 ± 0.27	2.11	97.4	13.1 ± 0.12	0.90	101
	15	15.4 ± 0.14	0.91	96.1	15.7 ± 0.22	1.39	98.0	16.1 ± 0.19	1.17	100
5-Hydroxy-l-lys, 1st peak	0–100	*y* = 0.994*x* + 0.337, *r*^2^ = 0.9988			*y* = 0.9496*x* + 2.31, *r*^2^ = 0.9996			*y* = 0.959*x* + 3.32, *r*^2^ = 0.9992		
*m*/*z* 448 *m*/*z* 451 (IS, 40 µM)	0	1.93 ± 0.067	3.44	n.a	2.12 ± 0.03	1.53	n.a	2.24 ± 0.19	8.33	n.a
	20	19.8 ± 0.31	1.54	89.6	20.9 ± 0.67	3.19	93.8	22.3 ± 0.45	2.01	100
	40	38.8 ± 0.95	2.44	92.3	41.1 ± 0.82	1.99	97.3	41.7 ± 0.8	1.87	101
	60	58.7 ± 2.11	3.60	94.5	59 ± 2.94	4.97	94.8	60.9 ± 1.32	2.16	97.7
	80	79.7 ± 1.38	1.73	97.2	79.2 ± 3.59	4.53	96.3	79.5 ± 2.95	3.71	96.6
	100	101 ± 1.17	1.15	99.2	96.5 ± 3.02	3.13	94.4	97.2 ± 4.02	4.14	94.9
5-Hydroxy-l-Lys, 2nd peak	0–100	*y* = 0.984*x* + 9.78, *r*^2^ = 0.9995			*y* = 0.939*x* + 12.4, *r*^2^ = 0.9987			*y* = 0.932*x* + 14.6, *r*^2^ = 0.9972		
*m*/*z* 448 *m*/*z* 451 (IS, 40 µM)	0	10.8 ± 0.33	3.06	n.a	11.8 ± 0.25	1.53	n.a	13 ± 0.89	6.86	n.a
	20	28.8 ± 0.72	2.51	90.0	30.5 ± 1.15	3.19	93.8	33 ± 0.54	1.63	99.9
	40	48.1 ± 0.96	1.99	93.3	50.6 ± 0.95	1.99	97.3	53.1 ± 1.45	2.72	100
	60	69.2 ± 2.02	2.91	97.4	70.9 ± 2.52	4.97	94.8	73.5 ± 1.91	2.60	101
	80	88.0 ± 1.29	1.46	96.4	88 ± 3.58	4.53	96.3	89 ± 2.45	2.75	95.0
	100	109 ± 2.21	2.03	98.0	105 ± 3.71	3.13	94.4	106 ± 4.63	4.38	92.8
l-Arginine	0–137.5	*y* = 1.01*x* + 17.9, *r*^2^ = 0.9993			*y* = 0.981*x* + 18.03, *r*^2^ = 0.9999			*y* = 1.041*x* + 18.4, *r*^2^ = 0.9998		
*m*/*z* 586 *m*/*z* 589 (IS, µM)	0	17 ± 0.68	4.00	n.a	18.5 ± 1.47	7.94	n.a	19.2 ± 0.47	2.42	n.a
	27.5	44.9 ± 0.68	1.51	102	44.4 ± 1.11	2.50	94.2	46.7 ± 0.7	1.50	100
	55	75.2 ± 0.43	0.57	106	71.4 ± 1.5	2.10	96.2	74.5 ± 1.4	1.87	100
	82.5	102 ± 1.17	1.15	103	100 ± 1.52	1.53	98.3	104 ± 0.19	0.19	102
	110	130 ± 0.87	0.67	103	127 ± 1.97	1.56	98.2	134 ± 1.2	0.90	104
	137.5	155 ± 1.17	0.76	100	152 ± 0.45	0.29	97.4	161 ± 3.06	1.89	103
*N*^G^-(2-Carboxyethyl)-l-Arg	0–25	*y* = 0.766*x* + 4.98, *r*^2^ = 0.9997			*y* = 0.773*x* + 4.99, *r*^2^ = 0.9991			*y* = 0.833*x* + 1.997, *r*^2^ = 0.9989		
*m*/*z* 514 *m*/*z* 517 (IS, µM)	0	4.92 ± 0.35	7.11	n.a	5.03 ± 0.22	4.38	n.a	5.15 ± 0.096	1.86	n.a
	5	8.84 ± 0.26	2.99	78.4	8.87 ± 0.27	2.99	76.8	9.03 ± 0.28	3.11	77.5
	10	12.7 ± 0.58	4.54	77.6	12.4 ± 0.1	0.78	73.5	13.3 ± 0.13	1.01	81.8
	15	16.4 ± 0.21	1.29	76.3	16.9 ± 0.51	3.03	79.3	17.6 ± 0.75	4.28	82.9
	20	20.5 ± 0.62	3.02	78.0	20.5 ± 0.6	2.91	77.3	21.2 ± 0.3	1.43	80.4
	25	24 ± 0.07	0.27	76.3	24.2 ± 0.56	2.33	76.6	26.1 ± 0.28	1.07	84.0
*N*^G^-Monomethyl-l-Arg	0–5	*y* = 0.956*x* + 0.055, *r*^2^ = 0.9992			*y* = 0.984*x* + 0.0469, *r*^2^ = 0.9995			*y* = 1.011*x* + 0.03, *r*^2^ = 0.9979		
*m*/*z* 474 *m*/*z* 477 (IS, µM)	0	0.1 ± 0.01	9.73	n.a	0.1 ± 0.008	8.28	n.a	0.11 ± 0.008	7.43	n.a
	1	1.02 ± 0.06	5.91	91.8	1.01 ± 0.009	0.93	91.3	1.05 ± 0.02	1.87	94.2
	2	1.94 ± 0.02	1.15	92.2	1.96 ± 0.025	1.27	92.9	1.99 ± 0.089	4.48	93.9
	3	2.84 ± 0.08	2.93	91.4	2.97 ± 0.026	0.86	95.7	2.92 ± 0.04	1.38	93.7
	4	3.89 ± 0.13	3.39	94.7	4.01 ± 0.085	2.11	97.8	4.13 ± 0.12	2.93	101
	5	4.89 ± 0.07	1.40	95.9	4.98 ± 0.15	2.97	97.7	5.15 ± 0.023	0.44	101
*N*^G^-(2-Carboxymethyl)-l-Arg	0–25	*y* = 0.917*x* + 1.01, *r*^2^ = 0.9993			*y* = 0.926*x* + 1.05, *r*^2^ = 0.9994			*y* = 0.933*x* + 1.103, *r*^2^ = 0.9997		
*m*/*z* 500 *m*/*z* 503 (IS, µM)	0	1.03 ± 0.06	5.97	n.a	1.07 ± 0.03	2.79	n.a	1.12 ± 0.043	3.81	n.a
	5	5.5 ± 0.26	4.74	89.4	5.71 ± 0.13	2.26	92.9	5.82 ± 0.14	2.44	94.0
	10	10.5 ± 0.61	5.77	94.6	10.3 ± 0.38	3.65	92.5	10.5 ± 0.45	4.22	94.2
	15	14.4 ± 0.38	2.67	89.3	14.6 ± 0.25	1.69	90.5	14.9 ± 0.86	5.80	91.6
	20	19.3 ± 1.06	5.49	91.2	19.9 ± 0.72	3.61	94.3	19.7 ± 0.64	3.28	92.7
	25	24.1 ± 0.14	0.57	92.1	24.1 ± 0.72	3.01	92.1	24.6 ± 0.62	2.53	93.9
*S*-Carboxymethyl-l-Cys	0–25	*y* = 0.762*x* + 0.066, *r*^2^ = 0.9837			*y* = 0.8999*x* + 0.26, *r*^2^ = 0.9943			*y* = 0.835*x* + 0.984, *r*^2^ = 0.9991		
*m*/*z* 104 *m*/*z* 107 (IS, 10 µM)	0	0.9 ± 0.06	6.58	n.a	0.95 ± 0.17	17.9	n.a	1.08 ± 0.031	2.85	n.a
	5	3.76 ± 0.29	7.79	57.1	4.8 ± 0.68	14.1	77.0	4.97 ± 0.19	3.88	77.8
	10	7.34 ± 0.91	12.4	64.4	8.56 ± 0.19	2.25	76.1	9.66 ± 0.63	6.53	85.8
	15	10.6 ± 1.55	14.6	64.5	13 ± 1.36	10.5	80.2	13.2 ± 1.4	10.6	81.0
	20	14.5 ± 0.62	4.27	68.0	18.3 ± 1.31	7.14	87.0	17.6 ± 0.47	2.66	82.4
	25	20.5 ± 3.22	15.7	78.3	23.4 ± 0.74	3.17	89.9	22 ± 1	4.55	83.9
*S*-(2-Carboxyethyl)-l-Cys	0–25	*y* = 0.893*x* + 4.04, *r*^2^ = 0.9997			*y* = 0.843*x* + 4.59, *r*^2^ = 0.999			*y* = 0.931*x* + 4.91, *r*^2^ = 0.9996		
*m*/*z* 278 *m*/*z* 281 (IS, 10 µM)	0	3.98 ± 0.25	6.24	n.a	4.88 ± 0.43	8.82	n.a	5.03 ± 0.34	6.71	n.a
	5	8.41 ± 0.54	6.48	88.5	8.52 ± 0.47	5.53	72.8	9.25 ± 0.26	2.81	84.5
	10	13.2 ± 0.23	1.76	91.7	12.9 ± 0.39	3.05	80.3	14.4 ± 0.09	0.61	93.9
	15	17.6 ± 0.2	1.15	90.6	17.4 ± 0.53	3.07	83.2	18.9 ± 0.45	2.38	92.5
	20	21.7 ± 0.19	0.88	88.7	21.2 ± 0.27	1.29	81.5	23.5 ± 0.16	0.69	92.3
	25	26.4 ± 0.3	1.15	89.5	25.9 ± 0.54	2.09	84.1	28.2 ± 0.46	1.63	92.5
*S*-(2-Succinyl)-l-Cys	0–25	*y* = 1.028*x* + 11.1, *r*^2^ = 0.9993			*y* = 0.911*x* + 11.4, *r*^2^ = 0.9989			*y* = 1.059*x* + 12.02, *r*^2^ = 0.9986		
*m*/*z* 405 *m*/*z* 414 (IS, 10 µM)	0	11.3 ± 0.32	2.84	n.a	11.7 ± 1.16	9.83	n.a	12.3 ± 0.37	2.99	n.a
	5	16.2 ± 0.37	2.26	98.9	15.5 ± 0.78	5.06	74.9	17.3 ± 0.2	1.17	99.5
	10	21.6 ± 0.34	1.59	103	20.3 ± 0.32	1.59	85.5	22 ± 0.68	3.10	96.2
	15	26.1 ± 0.13	0.50	98.8	25.2 ± 0.1	0.39	89.5	27.9 ± 0.15	0.53	104
	20	31.9 ± 0.44	1.37	103	29.6 ± 0.73	2.47	89.4	33.6 ± 0.31	0.92	106
	25	36.9 ± 0.46	1.25	103	34.2 ± 0.93	2.71	89.8	38.5 ± 0.78	2.03	104

*n.a.* not applicable

The precision of the method ranged between 0.27% and 17.9%. The accuracy of the method ranged between 76.3 and 112%. The lowest recovery values (76.2%, 90%, 83.5%) were obtained for CMC, notably on method validation on day #1. The lower recovery values for CMC could be in part due to the low intensity of the mass fragments used in the validation. The selected mass fragments were used to achieve higher specificity in quantitative measurements. The correlation coefficients (*r*^2^) ranged between 0.9837 and 0.999. The validation results indicate that the GC–MS method is useful for the precise and accurate measurement of low concentrations of the PTM metabolites and AGEs in the presence of considerably higher concentrations of the parent amino acids in human urine. The results of Table S1 confirm the validity of the GC–MS method for other amino acids (Hanff et al. 2019). Thus, the GC–MS method presented here is suitable for the simultaneous quantitative determination of amino acids and their PTM metabolites and AGEs.

In the human urine sample used in method validation, *S*-(2-succinyl)-l-Cys (S2C) and 5-hydroxy-l-Lys (5-OH-Lys, 2nd GC–MS peak) were found to be the most abundant metabolites followed by CML, CEL and CEA. It appears from Table 1 that the concentrations of the analytes in the un-spiked urine samples increased after 1 and 2 days (Table 4). Yet, the concentrations of all measured amino acids also increased compared to the first day of validation, on average by 5% on day #2 and by 11% on day #3 (Fig. S5).

Typical GC–MS chromatograms from the simultaneous quantitative analysis of amino acids, their PTM metabolites and AGEs in a human urine sample are shown in Fig. S6.

### Stability of the amino acid derivatives in toluene extracts

As toluene extracts may not always be analyzed immediately after derivatization and extraction by toluene (Baskal et al. 2021b), we randomly selected the toluene extracts of seven different urine samples and analyzed by GC–MS freshly obtained toluene extracts (day #1). After completion of the first analysis run, the toluene extracts were analyzed again on next day (day #2) under the same GC–MS conditions. Then, the autosampler samples were sealed again and left stand at room temperature until renewed analysis one week later (day #8). This procedure was repeated one more time and the toluene extracts were analyzed again one week later (day #15). Statistical analysis of the concentrations of the analytes revealed reproducible results for Lys, Arg, and their PTM metabolites and AGEs (Table S2). The peak areas of the endogenous analytes and their internal standards did not change remarkably suggesting that the amino acid derivatives are stable for at least two weeks, thus allowing reliable quantitative determination in human urine samples at least within two weeks after sample derivatization. The highest reproducibility values were observed for 5-hydroxy-lysine, lysine, monomethyl-lysine, arginine, ADMA, succinyl-cysteine and furosine (relative standard deviation, RSD, 1.5–4.7%), with the other analytes showing poorer yet acceptable reproducibility (RSD, 8.9–15.2%) (Table S2).

### Amino acids and their PTM metabolites and AGEs in boys’ urine of the ASOS study

The validated GC–MS method was applied to measure the concentration of PTM metabolites and AGEs of Lys, Arg and Cys as well as of other amino acids in urine samples of 39 black boys and 41 white boys of the ASOS study. As the urine samples were collected by spontaneous micturition, we also measured the creatinine concentrations in the ASOS urine samples, and corrected the urinary excretion of the amino acids by the respective creatinine concentration. The results of these analyses are summarized in Table 5. The urinary creatinine concentrations did not differ between black and white boys (15.3 [10.1–21.4] mM vs. 15.9 [12.8–18.8] mM, *P* = 0.504).

**Table 5:** Creatinine-corrected urinary excretion rates of amino acids, of their PTM metabolites and AGEs of Lys, Arg and Cys in the black and white boys of the ASOS study as measured by GC–MS

Amino acid PTM, AGEs	Black boys (*n* = 39)	White boys (*n* = 41)	*P* (Mann–Whitney)
Ala	24 [18.1–33.1]	24.6 [17.7–29.1]	0.9637
**Thr**	26.1 [21.2–35.0]	30.5 [26–35.2]	**0.0467**^a^
Gly	91 [60–117]	80.4 [65–109]	0.5478
Val	3.64 [2.92–4.45]	3.64 [3.18–4.32]	0.8877
Ser	38.7 [29.3–43.7]	35.4 [29–39.5]	0.1331
Sarc	0.047 [0.038–0.056]	0.048 [0.039–0.052]	0.9752
Leu/Ile	5.88 [5.33–6.84]	6.15 [5.66–7.25]	0.2969
GAA	46.3 [29.4–62.2]	39.5 [29–45.7]	0.1165
**Asn/Asp**	35.4 [29.2–48.7]	30 [26.4–38.9]	**0.0484**
**OH-Pro**	0.309 [0.253–0.378]	0.278 [0.241–0.319]	**0.0296**
**Pro**	0.422 [0.369–0.461]	0.384 [0.335–0.433]	**0.0154**
Met	16.4 [12.5–19.6]	15 [12.7–17.4]	0.3174
Gln/Glu	167 [139–186]	165 [148–191]	0.8016
Orn/Cit	4.02 [3.36–4.53]	3.95 [3.56–4.62]	0.7648
Phe	5.99 [4.88–6.79]	5.6 [4.67–6.94]	0.4534
Tyr	14.5 [11.1–16.6]	13.7 [12.3–15.7]	0.4918
Lys	10.1 [8.13–12.9]	11.6 [9.19–16.7]	0.1045
**Arg**	4.17 [3.36–4.74]	3.44 [2.84–4.08]	**0.0129**
**MML**	0.33 [0.20–0.63]	0.99 [0.54–1.79]	** < 0.0001**
**CMC**	0.188 [0.145–0.214]	0.28 [0.23–0.33]	** < 0.0001**
**hArg**	0.193 [0.135–0.367]	0.308 [0.173–0.459]	**0.0484**
CEL	0.919 [0.717–1.33]	0.96 [0.86–1.17]	0.6001
Trp	4.56 [3.65–6.19]	5.03 [4.06–6.65]	0.3060
CML	1.49 [0.99–1.84]	1.41 [1.15–1.88]	0.3791
ADMA	5.8 [5.4–7.1]	5.81 [5.16–6.84]	0.3766
CEC	1.36 [0.82–1.81]	1.21 [0.92–1.63]	0.8651
S2C	1.95 [1.75–2.3]	1.93 [1.69–2.19]	0.6823
CEA	3.02 [1.38–4.17]	2.44 [1.98–3.42]	0.8501
MMA	0.025 [0.016–0.032]	0.019 [0.015–0.025]	0.0713
Furosine	0.203 [1.32–0.283]	0.233 [0.16–0.366]	0.0573
CMA	0.332 [0.228–0.603]	0.30 [0.24–0.56]	0.9256
**5-OH-Lys_D**	0.65 [0.46–0.84]	0.55 [0.40–0.61]	**0.0030**
5-OH-Lys_L	4.81 [3.95–5.42]	4.51 [3.9–6.02]	0.4744
5-OH-Lys_L/D	15.4 [10–21.5]	15.8 [12.8–18.6	0.5336
Pentosidine	0.399 [0.229–0.535]	0.333 [0.230–0.426]	0.211

Pentosidine, a common AGE of Lys and Arg, was measured by ELISA (Mokwatsi et al. 2017)

^a^Bold *P* values indicate statistical significance

Statistically significant differences between black and white boys were found for the creatinine-corrected excretions of Thr and hArg (lower in the blacks), Asn/Asp, Pro, and Arg (higher in the blacks). The excretion rates of OH-Pro and 5-OH-Lys-D were higher in the black compared to the white boys. The excretion rates of MML and CMC were lower in the black compared to the white boys. The greatest differences between the groups were found for MML (threefold in the whites). ROC curve analyses showed that of all analytes (range 0.50–0.66) only CMC and MML were associated with high area values: 0.840 for CMC and 0.796 for MML with *P* < 0.0001 (Fig. S7). The excretion rates of furosine and MMA failed significant differences (Table 5). Only in the white group, a single correlation between the urinary excretion rate of hArg and age was found (*r* = 0.375, *P* = 0.016).

## Discussion

We developed and validated a stable-isotope dilution GC–MS method for the measurement of PTM metabolites and AGEs of proteinogenic amino acids in human urine. This method is based on our previous experience on the GC–MS analysis of proteinogenic and non-proteinogenic amino acids, including the *N*^G^-methylated metabolites of Arg originating from its PTM, in biological samples (Tsikas et al. 2003; Kayacelebi et al. 2014; Hanff et al. 2019), The focus of the present study was the methylation and glycation of the terminal *N*^ε^ and *N*^G^ groups of Lys and Arg, respectively, and the sulfhydryl (SH) group of Cys. Upon validation in human urine, we used the GC–MS method to study potential differences in amino acids metabolism in a bi-ethnic South African population, the ASOS study. The results of the present study are discussed below from analytical and biological perspectives.

### GC–MS analysis of urinary amino acids, their PTM metabolites and AGEs

The terminal groups of Lys and Arg in proteins undergo many PTMs, such as enzymatic methylation and chemical carboxymethylation and carboxyethylation. Because of the indispensable requirement of chemical derivatization of amino acids in GC–MS-based analytical methods, PTM-induced changes may require modifications of the derivatization procedures originally found optimal to the parent non-modified amino acids. Previously, we found that free and protein-incorporated ADMA is stable under the proteolysis and derivatization conditions (Hanff et al. 2019; Bollenbach et al. 2019, 2020b). In contrast, the isomer SDMA was found to be unstable (Bollenbach et al. 2018), suggesting that even structurally very similar amino acid metabolites may require different experimental conditions. In addition, the carbamoyl amino acids Cit, Gln and Asn are unstable under the conditions of esterification reaction (60 min, 80 °C) and undergo conversion to Orn, Glu and Asp, respectively (Hanff et al. 2019; Baskal et al. 2021c). Thus, while *N*^ε^-mono- and -dimethylated Lys are expected to be stable, the *N*^ε^-carboxymethylated and the *N*^ε^-carboxyethylated Lys, Arg and Cys, may decompose during esterification to release Lys, Arg and Cys, respectively. Because the concentration of proteins in human urine is very low and AGE residues in proteins are expected to be thermally labile, we resigned the classical proteolysis step, which requires drastic conditions (heating for 110 °C overnight in 6 M HCl).

We characterized structurally the Me-PFP derivatives of the PTM metabolites and AGEs. With exception of TML, all investigated amino acids had undergone the expected derivatization reactions. Because of its carboxylic and α-amino groups, TML is likely to have been converted to its Me-PFP derivative, which is however not extractable into toluene because of its terminal ammonium group. It is notable that TML can be analyzed by LC–MS/MS as its butyl ester (Schwedhelm et al. 2021). DML and MML were converted to their Me-PFP derivative(s), with the yield of the Me-PFP derivative of DML being relatively low. CML and CEL were found to partly decompose to form the Me-PFP derivative of Lys, suggesting loss of their carboxymethyl and carboxyethyl residues, respectively. Yet, this was fully compensated by their deuterium-labelled analogs. In the present study, we also investigated potential interference by *N*^α^-methyl-lysine and *N*^α^-acetyl-lysine. These amino acids were also converted to their Me-PFP derivatives, but they produced different mass spectra and were also separated gas chromatographically from the Me-PFP derivatives of *N*^ε^-methyl- and *N*^ε^-acetyl-lysine. In confirmation of previous observations (Hanff et al. 2019), we found that Asn was converted to Asp, Cit to Orn, and Gln to Glu. For these amino acids, the reported concentrations reflect the sum of Asn and Asp, Cit and Orn, and Gln and Glu. Also, because the Me-PFP derivatives of the isomeric and isobaric Leu and Ile were not separated chromatographically, the reported data correspond to the sum of Leu + Ile.

Although Cys can be measured by the present method (Hanff et al. 2019), special pre-analytical precaution steps are necessary to avoid its oxidation to cystine during sample collection and storage. The AGEs of Cys are stable enough in urine and do not need special precautions. We dispensed on the measurement of Cys to keep the GC–MS method more straightforward. In total, we quantitated simultaneously 33 analytes in only 10 µL aliquots of human urine samples. The results obtained in the validation experiments indicate an accurate and precise GC–MS method for the simultaneous quantitative measurement of the investigated amino acids alongside their PTM metabolites and AGEs in very small aliquots of human urine in relevant concentration ranges. This is of particular significance because the concentration of many amino acids in urine is two and even three orders of magnitude higher than their PTMs and AGEs.

### Urinary amino acids and their PTMs and AGEs in the ASOS study

The creatinine-corrected excretion rates of many amino acids did not differ between black and white boys of the ASOS study. Statistically significant differences of relatively low extent were found for Thr, Asn/Asp, Pro, Arg and hArg, which are involved in many different pathways. The most pronounced difference between the black and white boys was found for MML. On average, black boys excreted almost three times less MML in the urine than white boys. This difference is likely to be attributed to a higher mono-methylation of proteinic Lys most likely in histones, but its significance remains elusive. Among the AGEs, the greatest difference was found for CMC which was higher in the white compared to black boys (by about 33%). These observations are in line with the ROC curve data of CMC (0.840, *P* < 0.0001) and MML (0.796, *P* < 0.0001). Other AGEs including CEL, CML, CEC and furosine (in this study) and pentosidine (Mokwatsi et al. 2017) did not differ between the two groups.

Of interest may be OH-Pro and OH-Lys, of which the median excretion rates were higher in the blacks compared to the whites, albeit each to a relatively low extent of 10% and 15%, respectively. These differences may indicate a higher activity of prolyl-hydroxylase and 5-lysyl-hydroxylase activity in the black boys, respectively. Both 4-OH-Pro and 5-OH-Lys are derived to a major part from PTM by oxidation of Pro and Lys residues in collagen, respectively (Gjaltema and Bank 2017; Wu 2020). Similar results were obtained for OH-Pro in urine of young black and white men, which were moreover inversely associated with central systolic blood pressure in the black men only (Mels et al. 2019).

### Comparison of urinary amino acid AGEs in children and adults

There are few reports on the urinary excretion of selected AGEs, notably, CML, CEL and furosine, in children and adults. The reported concentrations are comparable to those we measured in the children of the ASOS study.

By means of ELISA, the mean CML concentration in the urine of very young neonates (≤ days) was found to be 887 ng/mL corresponding to an approximate creatinine-corrected excretion rate of 0.9 µM/mM (Dittrich et al. 2006). The CML concentration in eight different commercially available infant formulas administered to the infants was found to range between 514 ng/mL (2.5 µM) and 11,372 ng/mL (55.7 µM) in that study (Dittrich et al. 2006). These results suggest that the mode of delivery has a greater influence on urinary CML excretion than the nutrition.

Chloroformate derivatization is widely used in amino acids analysis by GC–MS (Hušek and Macek 1975; Hušek et al. 2016). By means of such a GC–MS method, the creatinine-corrected excretion rates of free CML and free CEL were measured to be on average 1.2 and 1.7 µM/mM, respectively, in children of whose the age had not been reported (Petrovič et al. 2005). These excretion rates are close to those we measured in the present study in the children of the ASOS study (age range 6–8 years). Other AGEs had not been analyzed in that study (Petrovič et al. 2005).

The intake and renal excretion of CML by healthy adolescent males (range 11–14 years) who took diet low (5.4 mg CML/day) and high (11.3 CML mg/day) in Maillard reaction products (MRP) was investigated by LC–MS/MS (Delgado-Andrade et al. 2012). The urinary CML excretion rate was reported to be 1.3 mg/day MRP-low diet and 1.6 mg/day in MRP-high diet. Thus, adolescents being on higher CML diet excreted a lower fraction of ingested CML than those being on lower CML diet (25% vs. 15%) (Delgado-Andrade et al. 2012).

The AGEs status has been investigated in 38 children (mean age 7.6 years) with autism spectrum disorder (ASD) and in 31 age-matched healthy children by LC–MS/MS (Anwar et al. 2018). In spot morning urine samples, ASD children had higher CML (33.1 vs. 26.2 nmol/mg creatinine) and CMA (1.78 vs. 1.46 nmol/mg creatinine) median concentrations than the controls, but lower median renal clearance values (0.8 vs. 1.6 µL/mg creatinine, *P* = 0.0011) (Anwar et al. 2018). It is worth mentioning that the creatinine-corrected excretion rates of amino acids were considerably higher in the ASD patients compared to controls (Anwar et al. 2018).

Free CML and CEL were measured by LC–MS/MS in urine of the CODAM study comprising 464 elderly subjects (mean age, 59 years) with 25% suffering from type 2 diabetes mellitus (Maasen et al. 2019). In this study, the median urinary excretion rates of CML and CEL ranged between 0.92 and 0.96 µM/mM, and between 0.50 and 0.53 µM/mM, respectively. The dietary intake by the subjects of the CODAM study ranged between 2.3 and 3.9 mg/day CML and 1.8 and 2.9 mg/day CEL (based on FFQ). These doses correspond to the amounts of CML and CEL produced endogenously by humans. This study found no association between glycemic load and urinary CML or CEL (Maasen et al. 2019).

### Study limitations and strengths

The present study is an extension of previous study (Hanff et al. 2019) to include PTM metabolites of Lys and AGEs of Lys, Arg and Cys. The GC–MS method allows in situ laboratory preparation of deuterium-labelled amino acids, of their PTM-derived metabolites and AGEs for use as internal standards. The present study confirms that the GC–MS method is not specific to Orn, Cit, Gln, Glu, Asn and Asp in part due to conversion of Cit to Orn, Gln to Glu, and Asn to Asp, and in part due to identical GC–MS behavior of Leu and Ile. Nevertheless, the method is useful to measure the sum of Leu and Ile, and in approximation the sum of Orn and Cit, Gln and Glu, and of Asn and Asp. This GC–MS method does not allow measurement of TML because of its permanent charge due to its ammonium feature. Yet, it is most likely that any other GC–MS would also fail to measure TML. Presently, the most useful method to measure TML seems to be LC–MS/MS (Schwedhelm et al. 2021). The number of the boys of the ASOS study is relatively small and may affect the outcome. As amino acids, their PTM metabolites and AGEs are present in foods, and nutrition was not closely controlled in the ASOS study, differences in nutrition between black and white boys, mainly in collagen-rich foods, may have contributed to the differences found in some amino acids and their metabolites. Yet, reported studies suggest that regular nutrition is unlikely to contribute considerably to endogenous AGEs both in children and in adults (Dittrich et al. 2006; Maasen et al. 2019). As the children of the ASOS study lived in comparable socio-economic environmental, nutrition is unlikely to have contribution to the differences observed for CMC (an AGE of Cys) and DML (a PTM metabolite of Lys) in the present study.

## Conclusion

A stable-isotope dilution GC–MS method was developed and validated for the reliable simultaneous measurement of *N*^ε^- and *N*^G^-methylated and advanced glycated end-products of Lys, Arg and Cys alongside their native non-modified free amino acids in 10 µL aliquots of human urine. The method was applied to quantitate potential ethnic-related differences in amino acids, their PTM metabolites and AGEs in 80 boys of South African ASOS study. The most remarkable differences between black and white boys were found for *N*^ε^-monomethyllysine (threefold lower in blacks), (*S*-carboxymethyl)-l-cysteine (1.5-fold lower in blacks) and 5-hydroxy-lysine (1.2-fold higher in blacks) suggesting ethnic-specific differences in chemical and enzymatic post-translational modifications of Lys- and Cys-containing proteins. The physiological importance of these ethnic-related differences for arterial stiffeness and vascular compliance remains to be elucidated.

## Supplementary Information

Below is the link to the electronic supplementary material.

Supplementary file1 (DOCX 586 KB)

## Abbreviations

ADMA: Asymmetric dimethylarginine
AGEs: Advanced glycated end-products
ASOS: Arterial Stiffness in Offspring Study
CEL: *N*^ε^-Carboxyethyllysine
CMC: *S*-(2-Carboxymethylcysteine)
CML: *N*^ε^-Carboxymethyllysine
DML: *N*^ε^*,N*^ε^-Dimethyllysine
GAA: Guanidinoacetate
GC–MS: Gas chromatography-mass spectrometry
hArg: L-Homoarginine
IQR: Interquartile range
IS: Internal standard
LMM: Low molecular mass
LOD: Limit of detection
LOQ: Limit of quantification
M: Molecular mass
Me: Methyl; methyl ester
MeOH: Methanol
MMA: Monomethylarginine
MML: *N*^ε^-Monomethyllysine
*m*/*z*: Mass-to-charge ratio
NICI: Negative-ion chemical ionization
NO: Nitric oxide
NOS: Nitric oxide synthase
PAR: Peak area ratio
PFP: Pentafluoropropionyl
PFPA: Pentafluoropropionic anhydride
PKMT: Protein lysine methyl transferase
PRMT: Protein arginine methyl transferase
PTM: Post-translational modification
SAM: *S*-Adenosylmethionine
SDMA: Symmetric dimethylarginine
S2C: *S*-(2-Succinyl)-l-cysteine
SIM: Selected-ion monitoring
S/N or SN: Signal-to-noise ratio
TLC: Thin-layer chromatography
TML: *N*^ε^,*N*^ε^,*N*^ε^-Trimethyllysine
*t*_R_: Retention time
