# Supplementary material for: Development, validation of a GC–MS method for the simultaneous measurement of amino acids, their PTM metabolites and AGEs in human urine, and application to the bi-ethnic ASOS study with special emphasis to lysine
Source: Amino Acids. 2021 Jul 12;54(4):615–41. doi: 10.1007/s00726-021-03031-6 (PMC9117344; doi:10.1007/s00726-021-03031-6)
Supplement: Supplementary file 1 — Supplementary file1 (DOCX 586 KB) [file 726_2021_3031_MOESM1_ESM.docx]

**Supplement**

**Development, Validation of a GC-MS Method for the Simultaneous Measurement of Amino Acids, their PTM Metabolites and AGEs in Human Urine, and Application to the Bi-ethnic ASOS Study with special Emphasis to Lysine**

**Svetlana Baskal^1^, Alexander Bollenbach^1^, Catharina Mels^2,3^, Ruan Kruger^2,3^, Dimitrios Tsikas^1^,***

^1^Institute of Toxicology, Core Unit Proteomics, Hannover Medical School, Hannover, Germany

^2^Hypertension in Africa Research Team (HART), North-West University, Potchefstroom, South Africa

^3^MRC Research Unit for Hypertension and Cardiovascular Disease, North-West University, Potchefstroom, South Africa

**Figure S1.** GC-MS spectra of the methyl ester pentafluoropropionyl derivatives of L-lysine and its metabolites analysed in the study

The following figures show the GC-MS spectra of the methyl ester (left panel) and trideutero-methyl ester (right panel) pentafluoropropionyl derivatives of pure amino acids (each 5 nmol) investigated in the present study. Inserts indicate the structure of L-lysine and its metabolites. *t*_R_, retention time (min); MM, molecular masses (MM). See Table 1 of the main article.

**
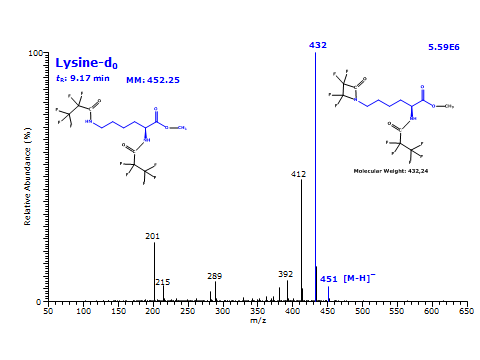

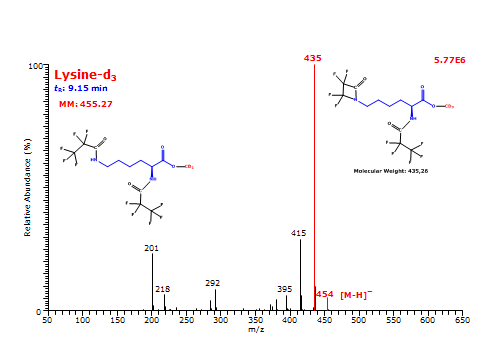
**

**Figure S2.** GC-MS spectra of the methyl ester pentafluoropropionyl derivatives of D,L-5-hydroxy-L-lysine

The following figures show the GC-MS spectra of the methyl ester (left panel) and trideutero-methyl ester (right panel) pentafluoropropionyl (PFP) derivatives of the commercially available D,L-5-hydroxy-L-lysine (each 5 nmol) investigated in the present study. The methyl ester-PFP derivative of D,L-5-Hydroxy-L-lysine eluted as an almost baseline-separated double GC-MS peak (see lowest panel). Inserts indicate the proposed structures of the derivatives and the mass fragments. *t*_R_, retention time (min); MM, molecular masses (MM). See Table 1 of the main article.

**
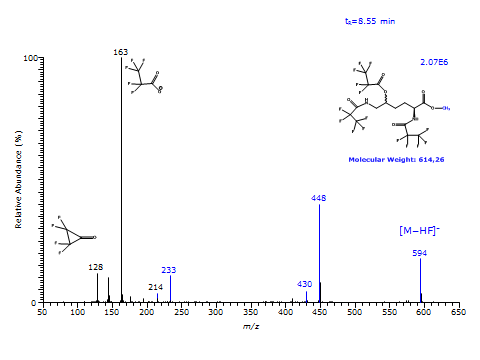

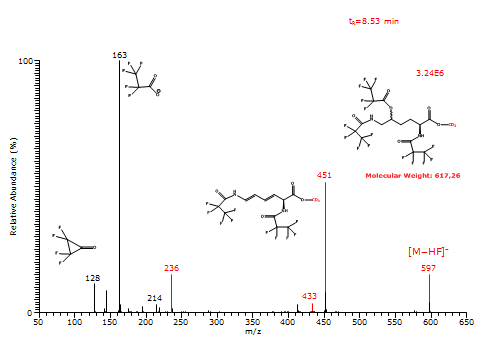
**

**
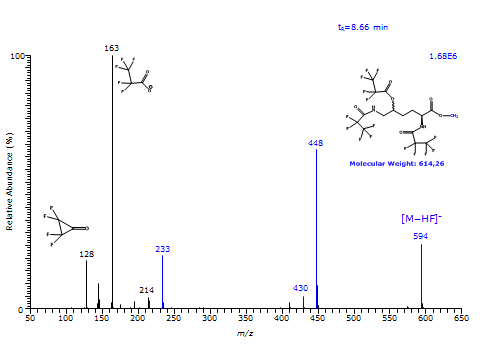

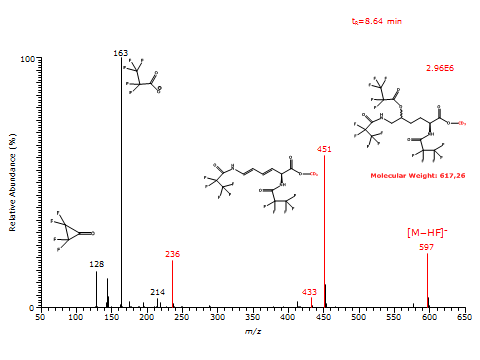
**

**
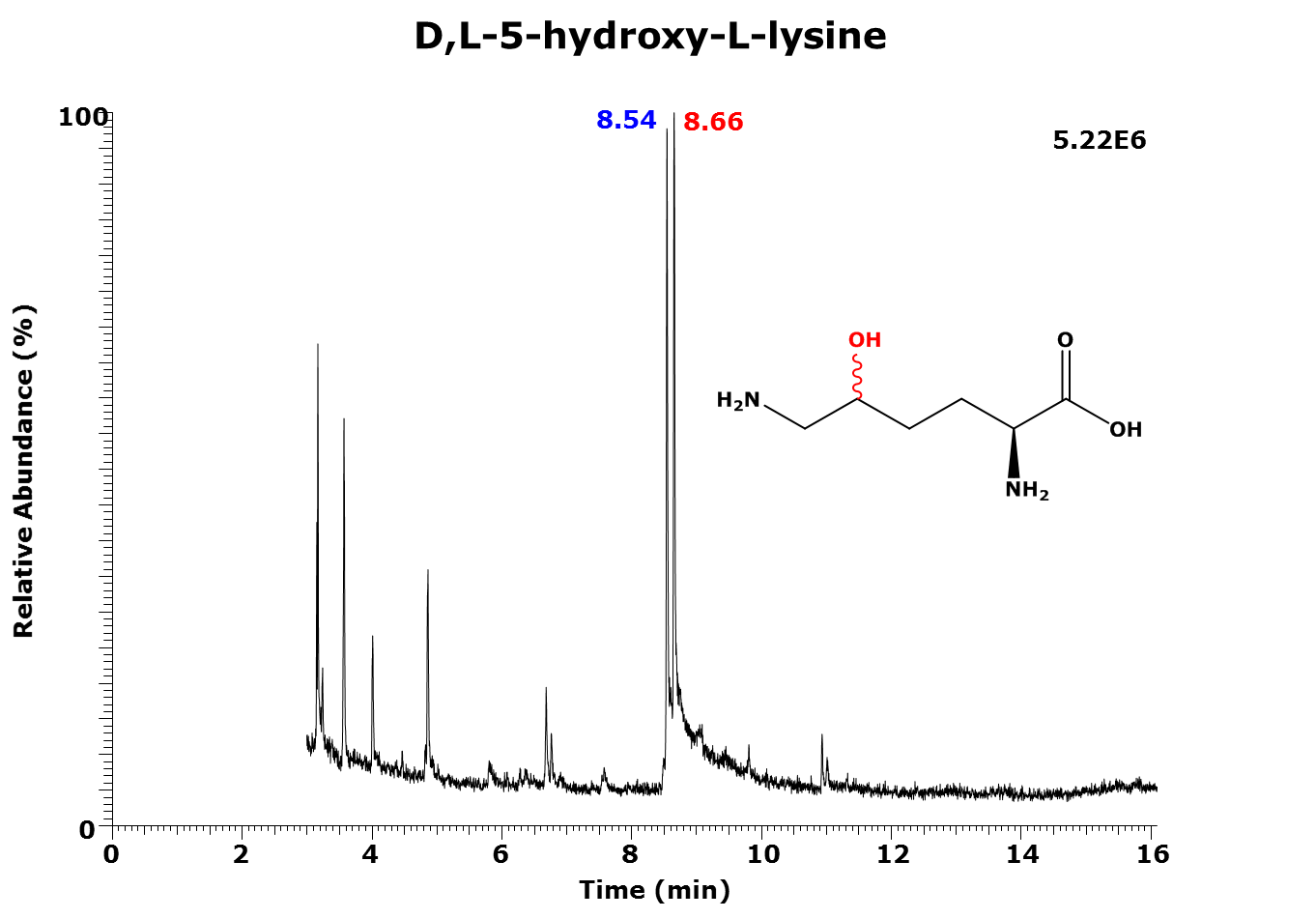
**

**Figure S3.** GC-MS spectra of the methyl ester pentafluoropropionyl derivates of (A) the unlabelled and (B) the deuterium-labelled of (*S*-carboxymethyl)-L-cysteine). The retention time was 10.0 min and 9.96 min, respectively. Insets indicate the proposed structures of the derivates and the mass fragments.

**
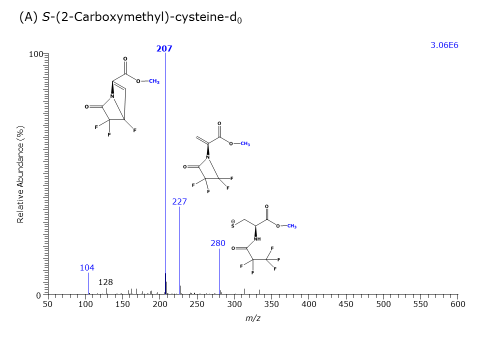

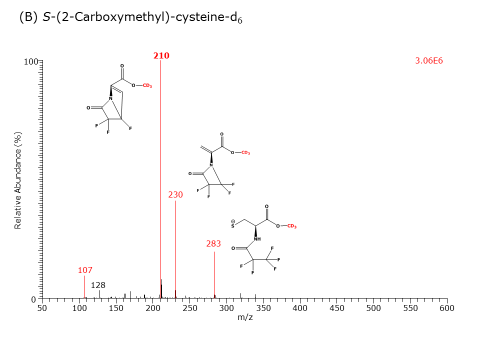
**

**Figure S4.** GC-MS spectra of the methyl ester pentafluoropropionyl derivates of (A) the unlabelled and (B) the deuterium-labelled of (*S*-carboxyethyl)-L-cysteine). The retention time was 11.83 min and 11.79 min, respectively. Insets indicate the proposed structures of the derivates and the mass fragments.

**
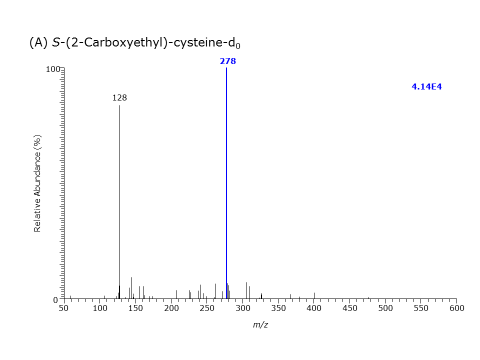
** **
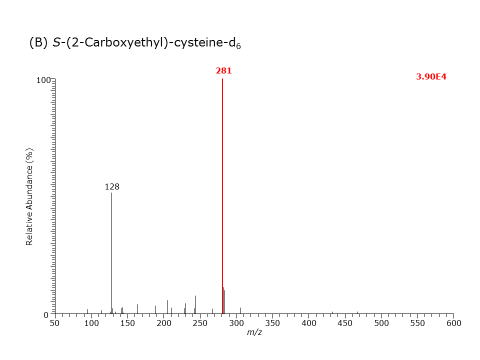
**

**Figure S5.** Stability of the indicated endogenous amino acids and their metabolites in the human urine sample used in method validation. See Table 5 in the main article for more details.

**Figure S6.** Total ion current chromatogram of endogenous amino acids (A, A’; upper panels, green) from the simultaneous quantitative analysis of a human urine sample and of the respective deuterium-labelled analogs (B, B’; lower panels, red) serving as the internal standards. Some peaks were magnified by a factor of 8. The *m/z* ratios of analytes and internal standards monitored and the time-windows are summarized in Table 3. The two peaks of 5-hydroxy-lysine are not shown. Note that some derivatives co-elute but are separated by mass spectrometry.

**
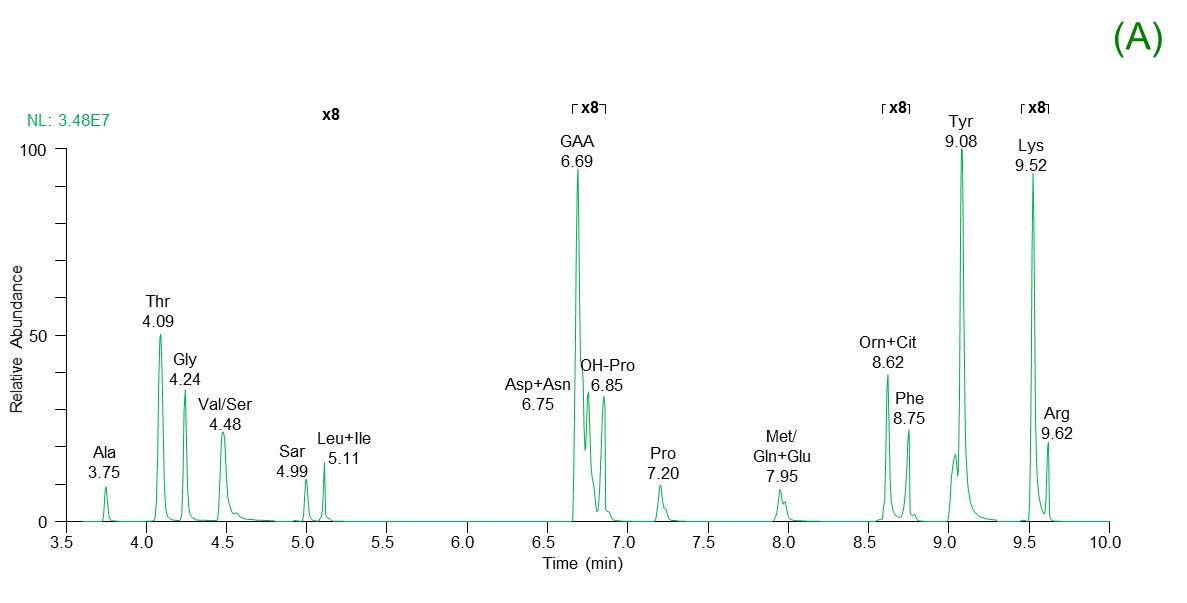
**

**
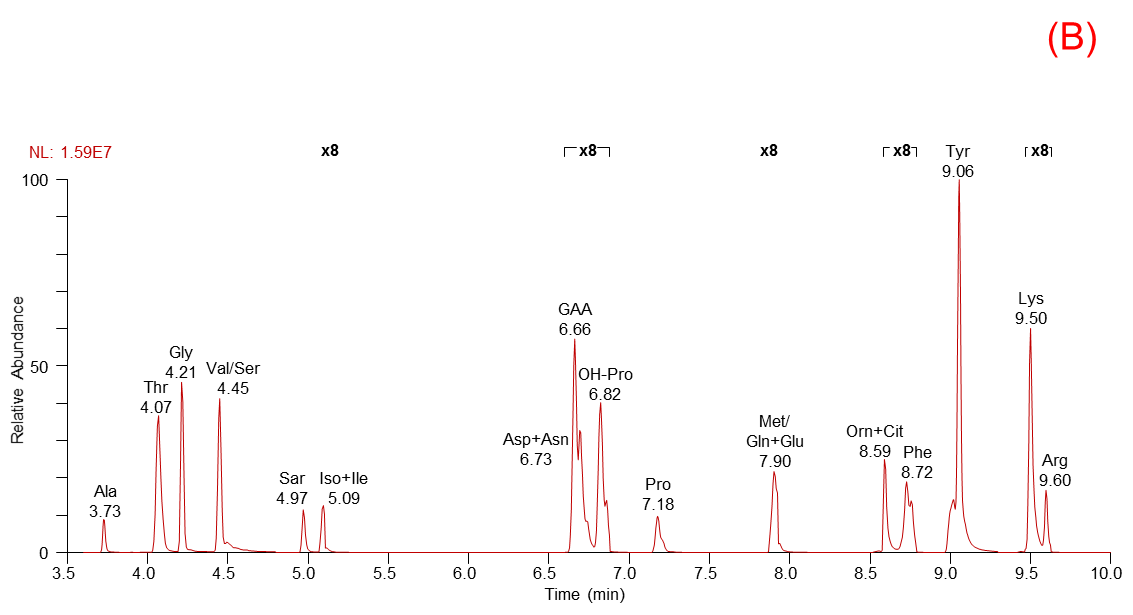
**

**
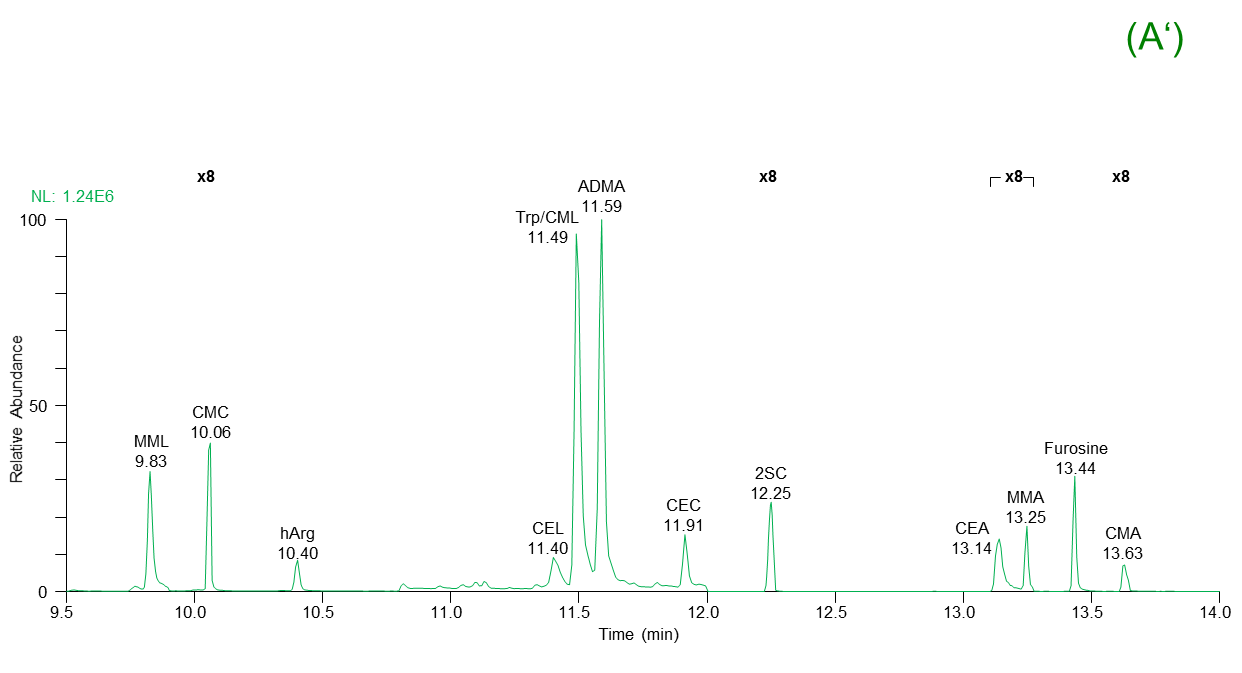
**


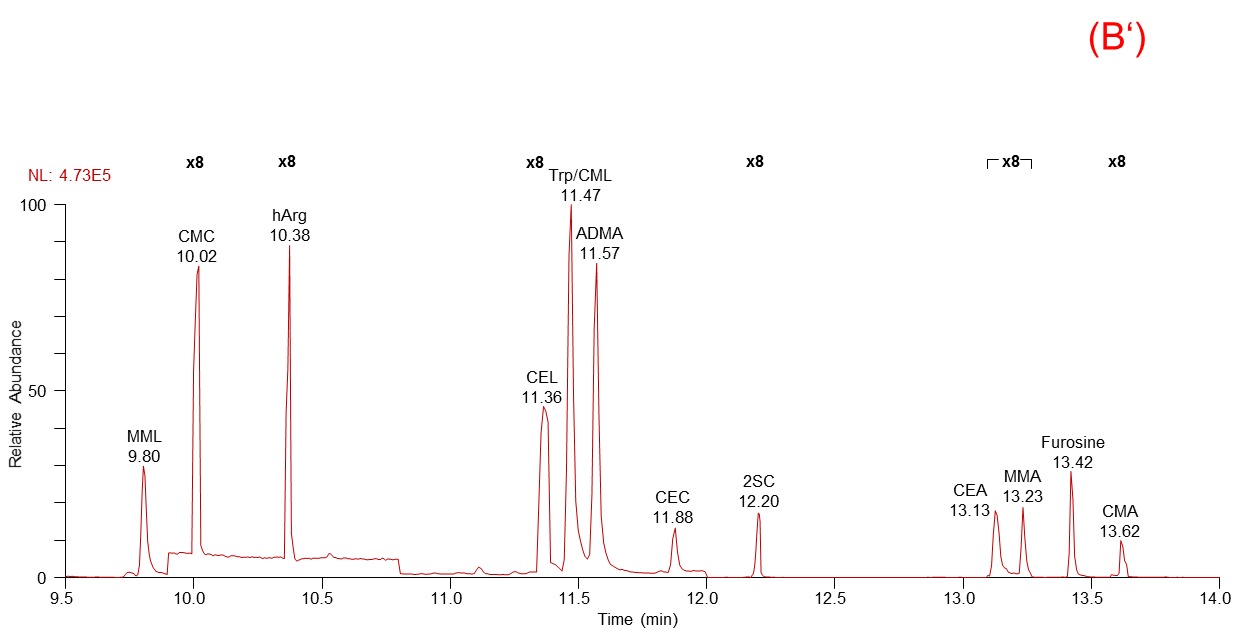


**Figure S7.** ROC curve for (A) *N*^ε^-monomethyllyseine and (B) *S*-(2-carboxymethylcysteine) in the urine samples of the ASOS study

Table S1. Summary of the results of the intra- and inter-day validation (precision, RSD) and accuracy (recovery) of the GC-MS method for the simultaneous measurement of Lys, Arg, Cys, and their PTM and AGE metabolites in human urine in the indicated biologically relevant concentration ranges using their deuterium-labelled methyl esters analogs as internal standards. n.a., not applicable

| **Amino acid derivative** | **Range (µM)** | **Day 1** |  |  | **Day 2** |  |  | **Day 3** |  |  |
| --- | --- | --- | --- | --- | --- | --- | --- | --- | --- | --- |
|  | Added  (µM) | Measured (µM) | RSD  (%) | Recovery  (%) | Measured  (µM) | RSD  (%) | Recovery  (%) | Measured  (µM) | RSD  (%) | Recovery  (%) |
| Me-**Ala**-(PFP) | **0-550** | y = 0.871x + 129, r^2^ = 0.9993 | | | y = 0.823x + 134, r^2^ = 0.9992 | | | y = 0.871x + 137, r^2^ = 0.9999 | | |
|  | 0 | 126 ± 4.61 | 3.64 | n.a. | 134 ± 11.8 | 8.79 | n.a. | 137 ± 1.9 | 1.39 | n.a. |
|  | 110 | 224 ± 4.76 | 2.12 | 88.9 | 220 ± 9.14 | 4.14 | 78.9 | 232 ± 1.2 | 0.52 | 86.1 |
|  | 220 | 327 ± 4.62 | 1.41 | 91.2 | 316 ± 3.1 | 0.98 | 83.1 | 327 ± 1.88 | 0.57 | 86.4 |
|  | 330 | 412 ± 8.24 | 2.00 | 86.6 | 409 ± 6.48 | 1.58 | 83.4 | 426 ± 7.39 | 1.74 | 87.3 |
|  | 440 | 518 ± 10.7 | 2.07 | 89.0 | 502 ± 14.1 | 2.81 | 83.7 | 522 ± 5.67 | 1.09 | 87.4 |
|  | 550 | 604 ± 4.14 | 0.69 | 86.8 | 580 ± 4.48 | 0.77 | 81.1 | 615 ± 5.31 | 0.86 | 86.8 |
| Me-**Thr**-(PFP) | **0-412.5** | y = 1.166x + 171, r^2^ = 0.9981 | | | y = 1.061x + 170, r^2^ = 0.9982 | | | y = 1.155x + 189, r^2^ = 0.9992 | | |
|  | 0 | 168 ± 13.4 | 7.92 | n.a. | 164 ± 9.58 | 5.86 | n.a. | 185 ± 1.66 | 0.90 | n.a. |
|  | 82.5 | 267 ± 6.95 | 2.60 | 120 | 259 ± 6.58 | 2.54 | 116 | 286 ± 1.87 | 0.65 | 122 |
|  | 165 | 377 ± 9.89 | 2.62 | 127 | 348 ± 6.59 | 1.90 | 112 | 383 ± 6.22 | 1.63 | 120 |
|  | 247.5 | 450 ± 38.6 | 8.59 | 114 | 443 ± 5.44 | 1.23 | 113 | 477 ± 6.42 | 1.35 | 118 |
|  | 330 | 554 ± 4.5 | 0.81 | 117 | 523 ± 1.61 | 0.31 | 109 | 577 ± 8.17 | 1.42 | 119 |
|  | 412.5 | 655 ± 9.05 | 1.38 | 118 | 599 ± 10.3 | 1.72 | 106 | 658 ± 8.37 | 1.27 | 115 |
| Me-**Gly**-(PFP) | **0-2750** | y = 0.636x + 701, r^2^ = 0.9909 | | | y = 0.574x + 712, r^2^ = 0.9891 | | | y = 0.607x + 676, r^2^ = 0.9978 | | |
|  | 0 | 639 ± 43.5 | 6.80 | n.a. | 671 ± 70.6 | 10.5 | n.a. | 636 ± 17.8 | 2.80 | n.a. |
|  | 550 | 1062 ± 45.4 | 4.27 | 76.8 | 1020 ± 60.1 | 5.89 | 63.5 | 1025 ± 38.2 | 3.73 | 70.7 |
|  | 1100 | 1463 ± 50.1 | 3.42 | 74.9 | 1386 ± 58.5 | 4.22 | 65.0 | 1379 ± 39.3 | 2.85 | 67.5 |
|  | 1650 | 1756 ± 81.8 | 4.66 | 67.7 | 1669 ± 64.6 | 3.87 | 60.5 | 1700 ± 32.9 | 1.94 | 64.5 |
|  | 2200 | 2167 ± 82.7 | 3.82 | 69.4 | 2061 ± 137 | 6.63 | 63.2 | 1997 ± 62.2 | 3.11 | 61.9 |
|  | 2750 | 2364 ± 62.5 | 2.64 | 62.7 | 2200 ± 70.7 | 3.21 | 55.6 | 2323 ± 42.9 | 1.85 | 61.4 |
| Me-**Val**-(PFP) | **0-82.5** | y = 0.86x + 33.9, r^2^ = 0.9553 | | | y = 0.645x + 33.8, r^2^ = 0.9895 | | | y = 0.635x + 34.4, r^2^ = 0.9987 | | |
|  | 0 | 32.2 ± 4.6 | 14.3 | n.a. | 34.6 ± 3.47 | 10.0 | n.a. | 34.3 ± 1.83 | 5.33 | n.a. |
|  | 16.5 | 48.3 ± 5.18 | 10.7 | 97.7 | 42.8 ± 3.98 | 9.30 | 49.8 | 44.6 ± 1.19 | 2.67 | 62.5 |
|  | 33 | 67.1 ± 6.65 | 9.91 | 106 | 55.6 ± 7.51 | 13.5 | 63.8 | 56.5 ± 3.11 | 5.49 | 67.4 |
|  | 49.5 | 69.2 ± 4.38 | 6.33 | 74.9 | 64.7 ± 4.42 | 6.83 | 60.8 | 65.2 ± 3.88 | 5.95 | 62.5 |
|  | 66 | 98.9 ± 13.2 | 13.3 | 101 | 79.8 ± 5.66 | 7.09 | 68.6 | 75.7 ± 8.82 | 11.6 | 62.8 |
|  | 82.5 | 100.7 ± 13.2 | 13.2 | 83.1 | 85 ± 9.9 | 11.7 | 61.1 | 87.3 ± 3.28 | 3.75 | 64.2 |
| Me-**Ser**-(PFP) | **0-825** | y = 0.728x + 325, r^2^ = 0.9818 | | | y = 0.6002x + 330, r^2^ = 0.9907 | | | y = 0.6003x + 350, r^2^ = 0.9919 | | |
|  | 0 | 308 ± 2.48 | 0.81 | n.a. | 316 ± 20.9 | 6.63 | n.a. | 329 ± 4.08 | 1.24 | n.a. |
|  | 165 | 450 ± 13.3 | 2.96 | 86.0 | 431 ± 20.8 | 4.83 | 70.0 | 452 ± 7.26 | 1.61 | 74.4 |
|  | 330 | 595 ± 33.4 | 5.61 | 87.1 | 547 ± 24 | 4.40 | 70.1 | 569 ± 12.9 | 2.26 | 72.8 |
|  | 495 | 654 ± 31.1 | 4.75 | 70.0 | 617 ± 24.9 | 4.04 | 60.9 | 659 ± 23.1 | 3.50 | 66.7 |
|  | 660 | 848 ± 101 | 11.9 | 81.8 | 749 ± 32.9 | 4.39 | 65.6 | 748 ± 58.2 | 7.78 | 63.5 |
|  | 825 | 899 ± 104 | 11.7 | 71.6 | 804 ± 72.9 | 9.06 | 59.3 | 826 ± 46.2 | 5.59 | 60.3 |
| Me-**Sar**-(PFP) | **0-13.75** | y = 0.918x + 0.322, r^2^ = 0.9988 | | | y = 0.9143x + 0.258, r^2^ = 0.9999 | | | y = 0.954x + 0.246, r^2^ = 0.9998 | | |
|  | 0 | 0.26 ± 0.01 | 3.97 | n.a. | 0.3 ± 0.011 | 3.69 | n.a. | 0.33 ± 0.011 | 3.42 | n.a. |
|  | 2.75 | 2.77 ± 0.004 | 0.15 | 91.3 | 2.74 ± 0.05 | 1.84 | 88.6 | 2.87 ± 0.036 | 1.26 | 92.5 |
|  | 5.5 | 5.38 ± 0.1 | 1.95 | 93.0 | 5.24 ± 0.023 | 0.44 | 89.9 | 5.39 ± 0.066 | 1.23 | 92.1 |
|  | 8.25 | 8.21 ± 0.49 | 6.02 | 96.3 | 7.79 ± 0.075 | 0.97 | 90.8 | 8.04 ± 0.13 | 1.58 | 93.4 |
|  | 11 | 10.4 ± 0.084 | 0.81 | 91.7 | 10.4 ± 0.19 | 1.81 | 91.7 | 10.8 ± 0.01 | 0.091 | 95.0 |
|  | 13.75 | 12.8 ± 0.14 | 1.08 | 91.3 | 12.8 ± 0.14 | 1.10 | 90.9 | 13.4 ± 0.14 | 1.07 | 95.2 |
| Me-**Leu**-(PFP) | **0-220** | y = 1.104x + 36.3, r^2^ = 0.9992 | | | y = 1.055x + 35.8, r^2^ = 0.9988 | | | y = 1.117x + 37.3, r^2^ = 0.9993 | | |
|  | 0 | 37.9 ± 0.96 | 2.52 | n.a. | 38.9 ± 2.82 | 7.25 | n.a. | 40.4 ± 0.72 | 1.79 | n.a. |
|  | 44 | 83.8 ± 1.48 | 1.77 | 104 | 80.6 ± 2.43 | 3.01 | 95.0 | 85.7 ± 0.94 | 1.09 | 103 |
|  | 88 | 134 ± 2.35 | 1.75 | 109 | 126 ± 3.03 | 2.41 | 98.6 | 133 ± 2.25 | 1.69 | 105 |
|  | 132 | 178 ± 3.85 | 2.17 | 106 | 173 ± 2.97 | 1.72 | 101 | 182 ± 3.28 | 1.80 | 107 |
|  | 176 | 234 ± 3.78 | 1.62 | 111 | 226 ± 6.08 | 2.69 | 106 | 234 ± 4.91 | 2.10 | 110 |
|  | 220 | 279 ± 5.19 | 1.86 | 110 | 267 ± 2.2 | 0.82 | 104 | 286 ± 3.88 | 1.36 | 112 |
| Me-**GAA**-(PFP)_2_ | **0-550** | y = 0.977x + 269, r^2^ = 0.9997 | | | y = 0.9194x + 274, r^2^ = 0.9986 | | | y = 1.039x + 290, r^2^ = 0.9992 | | |
|  | 0 | 270 ± 10.3 | 3.83 | n.a. | 285 ± 22 | 7.73 | n.a. | 298 ± 4.38 | 1.47 | n.a. |
|  | 110 | 373 ± 7.14 | 1.91 | 94.0 | 367 ± 23.1 | 6.28 | 75.0 | 401 ± 3.3 | 0.82 | 93.2 |
|  | 220 | 489 ± 2.62 | 0.54 | 99.9 | 469 ± 7.35 | 1.57 | 83.6 | 512 ± 6.15 | 1.20 | 97.1 |
|  | 330 | 588 ± 6.77 | 1.15 | 96.6 | 578 ± 7.14 | 1.23 | 89.0 | 627 ± 9.72 | 1.55 | 99.7 |
|  | 440 | 703 ± 6.35 | 0.90 | 98.4 | 679 ± 14.8 | 2.18 | 89.6 | 750 ± 6.19 | 0.83 | 103 |
|  | 550 | 804 ± 11.7 | 1.45 | 97.2 | 783 ± 4.39 | 0.56 | 90.7 | 866 ± 12.6 | 1.46 | 103 |
| Me_2_-**Asp**-(PFP)_2_ | **0-206.25** | y = 1.173x + 152, r^2^ = 0.9975 | | | y = 1.0123x + 157, r^2^ = 0.9922 | | | y = 1.145x + 161, r^2^ = 0.9962 | | |
|  | 0 | 154 ± 8.3 | 5.39 | n.a. | 165 ± 16.3 | 9.89 | n.a. | 168 ± 3.87 | 2.30 | n.a. |
|  | 41.25 | 198 ± 10.6 | 5.34 | 107 | 191 ± 14.3 | 7.47 | 62.8 | 204 ± 1.75 | 0.86 | 86.4 |
|  | 82.5 | 252 ± 10.4 | 4.11 | 119 | 235 ± 8.29 | 3.52 | 85.1 | 251 ± 3.94 | 1.57 | 99.6 |
|  | 123.75 | 291 ± 6.07 | 2.09 | 111 | 278 ± 3.88 | 1.39 | 91.5 | 298 ± 4.96 | 1.67 | 104 |
|  | 165 | 352 ± 16.2 | 4.60 | 120 | 332 ± 17.5 | 5.27 | 101 | 349 ± 7.72 | 2.21 | 110 |
|  | 206.25 | 393 ± 14.5 | 3.69 | 116 | 364 ± 7.31 | 2.01 | 96.4 | 402 ± 10 | 2.49 | 114 |
| Me-**OH-Pro**-(PFP) | **0-55** | y = 0.832x + 1.04, r^2^ = 0.999 | | | y = 0.819x + 1.17, r^2^ = 0.9978 | | | y = 0.846x + 1.002, r^2^ = 0.9995 | | |
|  | 0 | 0.44 ± 0.013 | 2.93 | n.a. | 0.48 ± 0.028 | 5.92 | n.a. | 0.48 ± 0.016 | 3.30 | n.a. |
|  | 11 | 10.2 ± 0.082 | 0.81 | 88.4 | 10.3 ± 0.23 | 2.28 | 89.2 | 10.7 ± 0.24 | 2.22 | 92.8 |
|  | 22 | 20.1 ± 0.19 | 0.96 | 89.4 | 19.7 ± 0.18 | 0.92 | 87.1 | 19.9 ± 0.25 | 1.25 | 88.1 |
|  | 33 | 28.9 ± 0.26 | 0.89 | 86.1 | 28.5 ± 0.27 | 0.94 | 84.8 | 29 ± 0.34 | 1.18 | 86.4 |
|  | 44 | 37.8 ± 0.23 | 0.60 | 84.9 | 38.2 ± 1.32 | 3.45 | 85.7 | 38.5 ± 0.43 | 1.12 | 86.4 |
|  | 55 | 46.2 ± 0.15 | 2.49 | 83.2 | 45 ± 0.89 | 1.98 | 81.0 | 47.1 ± 0.5 | 1.06 | 84.8 |
| Me-**Pro**-(PFP) | **0-55** | y = 0.922x + 1.18, r^2^ = 0.9999 | | | y = 0.913x + 1.15, r^2^ = 0.9998 | | | y = 0.934x + 1.04, r^2^ = 0.9998 | | |
|  | 0 | 1.22 ± 0.04 | 3.68 | n.a. | 1.3 ± 0.084 | 6.42 | n.a. | 1.32 ± 0.051 | 3.83 | n.a. |
|  | 11 | 11.1 ± 0.31 | 2.76 | 90.3 | 11 ± 0.17 | 1.55 | 88.4 | 11.3 ± 0.11 | 1.01 | 90.9 |
|  | 22 | 21.7 ± 0.24 | 1.11 | 93.2 | 21 ± 0.56 | 2.65 | 89.6 | 21.3 ± 0.35 | 1.67 | 90.8 |
|  | 33 | 31.3 ± 0.44 | 1.40 | 91.3 | 31.3 ± 0.63 | 2.01 | 90.9 | 31.6 ± 0.39 | 1.23 | 91.8 |
|  | 44 | 42 ± 0.32 | 0.77 | 92.7 | 41.8 ± 0.83 | 1.99 | 92.1 | 42.2 ± 0.18 | 0.43 | 92.9 |
|  | 55 | 51.7 ± 0.9 | 1.74 | 91.9 | 51.1 ± 0.42 | 0.82 | 90.5 | 52.7 ± 1 | 1.89 | 93.3 |
| Me-**Met**-(PFP) | **0-137.5** | y = 0.759x + 139, r^2^ = 0.9775 | | | y = 0.565x + 145, r^2^ = 0.9978 | | | y = 0.701x + 148, r^2^ = 0.9979 | | |
|  | 0 | 136 ± 11 | 8.10 | n.a. | 146 ± 10.1 | 6.94 | n.a. | 149 ± 1.8 | 1.20 | n.a. |
|  | 27.5 | 162 ± 8.48 | 5.22 | 94.6 | 159 ± 8.3 | 5.21 | 49.2 | 167 ± 3.03 | 1.81 | 63.9 |
|  | 55 | 186 ± 6.68 | 3.60 | 89.4 | 176 ± 3.82 | 2.17 | 55.5 | 183 ± 2.98 | 1.62 | 61.5 |
|  | 82.5 | 195 ± 1.61 | 0.83 | 71.1 | 192 ± 0.83 | 0.43 | 56.5 | 206 ± 1.58 | 0.77 | 68.7 |
|  | 110 | 231 ± 10.7 | 4.61 | 86.4 | 209 ± 8.36 | 3.99 | 57.8 | 225 ± 4.51 | 2.01 | 68.5 |
|  | 137.5 | 239 ± 9.04 | 3.78 | 74.7 | 221 ± 6.03 | 2.72 | 54.9 | 245 ± 3.23 | 1.32 | 69.6 |
| Me_2_-**Glu**-(PFP)_2_ | **0-687.5** | y = 1.195x + 891, r^2^ = 0.999 | | | y = 0.931x + 897, r^2^ = 0.9978 | | | y = 1.236x + 910, r^2^ = 0.9984 | | |
|  | 0 | 881 ± 19.7 | 2.24 | n.a. | 898 ± 75.6 | 8.42 | n.a. | 919 ± 11.3 | 1.23 | n.a. |
|  | 137.5 | 1059 ± 27 | 2.55 | 129 | 1024 ± 66.6 | 6.50 | 91.7 | 1080 ± 21.7 | 2.01 | 117 |
|  | 275 | 1238 ± 18.6 | 1.50 | 130 | 1141 ± 11.5 | 1.01 | 88.2 | 1241 ± 25.1 | 2.02 | 117 |
|  | 412.5 | 1380 ± 20.4 | 1.48 | 121 | 1301 ± 25.2 | 1.94 | 97.7 | 1421 ± 15.6 | 1.10 | 122 |
|  | 550 | 1546 ± 25.7 | 1.66 | 121 | 1412 ± 54.4 | 3.85 | 93.5 | 1571 ± 10.7 | 0.68 | 119 |
|  | 687.5 | 1711 ± 21.6 | 1.26 | 121 | 1529 ± 7 | 0.46 | 91.8 | 1778 ± 21.8 | 1.23 | 125 |
| Me-**Orn**-(PFP)_2_ | **0-68.75** | y = 0.932x + 28.4, r^2^ = 0.9995 | | | y = 0.876x + 28.2, r^2^ = 0.9993 | | | y = 0.937x + 28.9, r^2^ = 0.999 | | |
|  | 0 | 28.2 ± 1.15 | 4.08 | n.a. | 29.1 ± 2.3 | 7.90 | n.a. | 29.8 ± 0.8 | 2.69 | n.a. |
|  | 13.75 | 40.8 ± 0.93 | 2.29 | 91.1 | 39.4 ± 2.1 | 5.35 | 74.9 | 41.5 ± 0.14 | 0.34 | 85.0 |
|  | 27.5 | 55 ± 0.33 | 0.60 | 97.5 | 51.9 ± 0.83 | 1.60 | 82.9 | 54 ± 0.1 | 0.18 | 87.8 |
|  | 41.25 | 66.6 ± 0.16 | 0.24 | 92.9 | 64.4 ± 0.82 | 1.27 | 85.7 | 66.6 ± 0.69 | 1.03 | 89.2 |
|  | 55 | 79.8 ± 0.75 | 0.94 | 93.8 | 76.5 ± 1.63 | 2.14 | 86.3 | 81.1 ± 1.13 | 1.39 | 93.2 |
|  | 68.75 | 92.1 ± 0.2 | 0.21 | 93.0 | 88.6 ± 0.6 | 0.68 | 86.6 | 93.8 ± 1.41 | 1.50 | 93.0 |
| Me-**Phe**-(PFP) | **0-220** | y = 0.983x + 33.8, r^2^ = 0.9998 | | | y = 0.939x + 34.1, r^2^ = 0.9996 | | | y = 1.01x + 34.6, r^2^ = 0.9993 | | |
|  | 0 | 34.7 ± 1.12 | 3.22 | n.a. | 35.6 ± 1.92 | 5.41 | n.a. | 37.3 ± 0.46 | 1.24 | n.a. |
|  | 44 | 76.5 ± 1.31 | 1.72 | 95.0 | 74.3 ± 1.94 | 2.61 | 88.0 | 78.8 ± 1.02 | 1.30 | 94.4 |
|  | 88 | 121 ± 0.36 | 0.30 | 98.1 | 115 ± 2.09 | 1.82 | 90.1 | 121 ± 1.46 | 1.20 | 95.2 |
|  | 132 | 162 ± 2.37 | 1.47 | 96.2 | 158 ± 2.35 | 1.49 | 92.9 | 166 ± 1.57 | 0.95 | 97.2 |
|  | 176 | 207 ± 3.03 | 1.46 | 98.2 | 202 ± 5.35 | 2.65 | 94.3 | 212 ± 0.98 | 0.46 | 99.3 |
|  | 220 | 251 ± 2.89 | 1.15 | 98.3 | 240 ± 2.48 | 1.04 | 92.8 | 260 ± 4.99 | 1.92 | 101 |
| Me-**Tyr**-(PFP) | **0-275** | y = 0.817x + 101, r^2^ = 0.9981 | | | y = 0.751x + 103, r^2^ = 0.9973 | | | y = 0.781x + 109, r^2^ = 0.9996 | | |
|  | 0 | 99 ± 2.47 | 2.50 | n.a. | 102 ± 7.2 | 7.08 | n.a. | 107 ± 1.66 | 1.55 | n.a. |
|  | 55 | 146 ± 2.76 | 1.90 | 84.6 | 142 ± 6.22 | 4.37 | 73.7 | 152 ± 0.91 | 0.60 | 81.8 |
|  | 110 | 196 ± 2.12 | 1.08 | 87.8 | 186 ± 3.01 | 1.62 | 76.7 | 196 ± 2.32 | 1.18 | 81.4 |
|  | 165 | 236 ± 2.57 | 1.09 | 83.3 | 227 ± 3.39 | 1.49 | 75.7 | 239 ± 3.81 | 1.59 | 80.5 |
|  | 220 | 286 ± 8.1 | 2.83 | 84.9 | 274 ± 10.8 | 3.93 | 78.5 | 280 ± 8.15 | 2.91 | 78.8 |
|  | 275 | 321 ± 11.9 | 3.72 | 80.9 | 303 ± 10.5 | 3.46 | 73.3 | 322 ± 5.85 | 1.82 | 78.2 |
| Me-**Lys**-(PFP)_2_ | **0-275** | y = 0.989x + 66.7, r^2^ = 0.999 | | | y = 0.963x + 66.2, r^2^ = 0.9983 | | | y = 1.014x + 70.1, r^2^ = 0.9998 | | |
|  | 0 | 65.1 ± 2.93 | 4.50 | n.a. | 67.1 ± 5.26 | 7.85 | n.a. | 69.8 ± 1.04 | 1.49 | n.a. |
|  | 55 | 120 ± 3.43 | 2.85 | 100 | 116 ± 4.81 | 4.16 | 88.3 | 125 ± 0.91 | 0.73 | 100 |
|  | 110 | 179 ± 1.38 | 0.77 | 103 | 172 ± 2.32 | 1.34 | 95.8 | 183 ± 1.93 | 1.06 | 103 |
|  | 165 | 229 ± 5.35 | 2.34 | 99.0 | 226 ± 2.11 | 0.93 | 96.6 | 238 ± 2.67 | 1.12 | 102 |
|  | 220 | 289 ± 0.96 | 0.33 | 102 | 284 ± 8.33 | 2.93 | 98.8 | 296 ± 6.27 | 2.12 | 103 |
|  | 275 | 335 ± 5.8 | 1.73 | 98.0 | 326 ± 5.33 | 1.63 | 94.1 | 347 ± 4.6 | 1.33 | 101 |
| Me-**Arg**-(PFP)_3_ | **0-137.5** | y = 1.01x + 17.9, r^2^ = 0.9993 | | | y = 0.981x + 18.03, r^2^ = 0.9999 | | | y = 1.041x + 18.4, r^2^ = 0.9998 | | |
|  | 0 | 17 ± 0.68 | 4.00 | n.a. | 18.5 ± 1.47 | 7.94 | n.a. | 19.2 ± 0.47 | 2.42 | n.a. |
|  | 27.5 | 44.9 ± 0.68 | 1.51 | 102 | 44.4 ± 1.11 | 2.50 | 94.2 | 46.7 ± 0.7 | 1.50 | 100 |
|  | 55 | 75.2 ± 0.43 | 0.57 | 106 | 71.4 ± 1.5 | 2.10 | 96.2 | 74.5 ± 1.4 | 1.87 | 100 |
|  | 82.5 | 102 ± 1.17 | 1.15 | 103 | 100 ± 1.52 | 1.53 | 98.3 | 104 ± 0.19 | 0.19 | 102 |
|  | 110 | 130 ± 0.87 | 0.67 | 103 | 127 ± 1.97 | 1.56 | 98.2 | 134 ± 1.2 | 0.90 | 104 |
|  | 137.5 | 155 ± 1.17 | 0.76 | 100 | 152 ± 0.45 | 0.29 | 97.4 | 161 ± 3.06 | 1.89 | 103 |
| Me-**hArg**-(PFP)_3_ | **0-13.75** | y = 1.104x + 0.779, r^2^ = 0.9998 | | | y = 1.052x + 0.756, r^2^ = 0.9992 | | | y = 1.164x + 0.651, r^2^ = 0.9989 | | |
|  | 0 | 0.85 ± 0.04 | 4.66 | n.a. | 0.87 ± 0.069 | 7.92 | n.a. | 0.86 ± 0.041 | 4.75 | n.a. |
|  | 2.75 | 3.66 ± 0.05 | 1.32 | 102 | 3.58 ± 0.043 | 1.19 | 98.6 | 3.81 ± 0.014 | 0.37 | 107 |
|  | 5.5 | 6.94 ± 0.12 | 1.75 | 111 | 6.49 ± 0.007 | 0.12 | 102 | 6.93 ± 0.1 | 1.51 | 110 |
|  | 8.25 | 9.87 ± 0.07 | 0.74 | 109 | 9.26 ± 0.17 | 1.83 | 102 | 10.1 ± 1.23 | 2.31 | 111 |
|  | 11 | 12.9 ± 0.1 | 0.81 | 110 | 12.6 ± 0.24 | 1.89 | 106 | 13.3 ± 0.14 | 1.05 | 113 |
|  | 13.75 | 16 ± 0.38 | 2.38 | 110 | 15.2 ± 0.2 | 1.33 | 104 | 16.9 ± 0.36 | 2.15 | 117 |
| Me-**Trp**-(PFP)_2_ | **0-137.5** | y = 0.8499x + 20.4, r^2^ = 0.9999 | | | y = 0.846x + 20.8, r^2^ = 0.999 | | | y = 0.915x + 21.3, r^2^ = 0.9992 | | |
|  | 0 | 20.3 ± 0.45 | 2.24 | n.a. | 21.7 ± 1.44 | 6.66 | n.a. | 22.5 ± 0.28 | 1.25 | n.a. |
|  | 27.5 | 43.8 ± 2.07 | 4.73 | 85.2 | 43.2 ± 0.82 | 1.90 | 78.1 | 46.7 ± 1.01 | 2.15 | 87.9 |
|  | 55 | 67.9 ± 0.95 | 1.40 | 86.4 | 66 ± 1.84 | 2.79 | 80.6 | 70.2 ± 0.71 | 1.01 | 86.6 |
|  | 82.5 | 90.2 ± 2.73 | 3.03 | 84.6 | 92.8 ± 3.26 | 3.51 | 86.2 | 94.9 ± 1.7 | 1.79 | 87.7 |
|  | 110 | 113 ± 2.72 | 2.40 | 84.5 | 113 ± 0.78 | 0.69 | 82.9 | 123 ± 1.22 | 0.99 | 91.3 |
|  | 137.5 | 138 ± 2.4 | 1.74 | 85.4 | 137 ± 1.93 | 1.40 | 84.1 | 148 ± 1.48 | 1.00 | 91.3 |
| Me-**ADMA**-(PFP)_3_ | **0-55** | y = 0.838x + 23.9, r^2^ = 0.9996 | | | y = 0.766x + 24.4, r^2^ = 0.9975 | | | y = 0.865x + 25.7, r^2^ = 0.9984 | | |
|  | 0 | 23.6 ± 0.24 | 3.13 | n.a. | 25.3 ± 1.5 | 5.91 | n.a. | 26.4 ± 0.56 | 2.12 | n.a. |
|  | 11 | 33.2 ± 0.65 | 1.95 | 87.6 | 32.1 ± 1.58 | 4.94 | 61.5 | 34.7 ± 0.17 | 0.50 | 74.8 |
|  | 22 | 42.7 ± 0.66 | 1.54 | 86.8 | 40.5 ± 0.87 | 2.16 | 69.1 | 44.1 ± 0.71 | 1.61 | 80.1 |
|  | 33 | 52.1 ± 0.39 | 0.76 | 86.4 | 50.5 ± 1.39 | 2.75 | 76.5 | 53.9 ± 0.69 | 1.29 | 83.1 |
|  | 44 | 60.5 ± 1.17 | 1.94 | 83.9 | 57.6 ± 0.7 | 1.22 | 73.4 | 64.8 ± 0.37 | 0.57 | 87.1 |
|  | 55 | 69.8 ± 0.8 | 1.15 | 84.1 | 66.9 ± 1.47 | 2.19 | 75.7 | 73 ± 0.21 | 0.28 | 84.6 |
| Me-**MMA**-(PFP)_2_ | **0-5** | y = 0.956x + 0.055, r^2^ = 0.9992 | | | y = 0.984x + 0.0469, r^2^ = 0.9995 | | | y = 1.011x + 0.03, r^2^ = 0.9979 | | |
|  | 0 | 0.1 ± 0.01 | 9.73 | n.a. | 0.1 ± 0.008 | 8.28 | n.a. | 0.11 ± 0.008 | 7.43 | n.a. |
|  | 1 | 1.02 ± 0.06 | 5.91 | 91.8 | 1.01 ± 0.009 | 0.93 | 91.3 | 1.05 ± 0.02 | 1.87 | 94.2 |
|  | 2 | 1.94 ± 0.02 | 1.15 | 92.2 | 1.96 ± 0.025 | 1.27 | 92.9 | 1.99 ± 0.089 | 4.48 | 93.9 |
|  | 3 | 2.84 ± 0.08 | 2.93 | 91.4 | 2.97 ± 0.026 | 0.86 | 95.7 | 2.92 ± 0.04 | 1.38 | 93.7 |
|  | 4 | 3.89 ± 0.13 | 3.39 | 94.7 | 4.01 ± 0.085 | 2.11 | 97.8 | 4.13 ± 0.12 | 2.93 | 101 |
|  | 5 | 4.89 ± 0.07 | 1.40 | 95.9 | 4.98 ± 0.15 | 2.97 | 97.7 | 5.15 ± 0.023 | 0.44 | 101 |

Table S2. Stability of the methyl ester pentafluoropropionyl derivatives in toluene extracts of Lys, Arg and their PTM metabolites and AGEs from urine samples of seven subjects analyzed by GC-MS on four days (#1, #2, #8 and #15). The numbers refer to the concentrations in the urine samples

| Urine sample | #1 | | #2 | | #3 | | #4 | | #5 | | #6 | | #7 | | All samples (mean±SD) | |
| --- | --- | --- | --- | --- | --- | --- | --- | --- | --- | --- | --- | --- | --- | --- | --- | --- |
| Amino acid | (µM) | (RSD) | (µM) | (RSD) | (µM) | (RSD) | (µM) | (RSD) | (µM) | (RSD) | (µM) | (RSD) | (µM) | (RSD) | (µM) | (RSD) |
| 5-OH-Lys (1^st^ peak) | 0.33 | 0.0 | 19.1 | 19.6 | 2.77 | 2.92 | 6.76 | 6.82 | 2.41 | 2.31 | 3.89 | 3.85 | 3.83 | 3.75 | 5.58±6.27 | 5.60±6.48 |
| 5-OH-Lys (2^nd^ peak) | 2.29 | 2.34 | 3.11 | 3.05 | 3.41 | 3.45 | 3.18 | 3.11 | 3.71 | 3.77 | 3.69 | 3.79 | 4.71 | 4.6 | 3.44±0.74 | 3.44±0.71 |
| Lys | 2.50 | 2.45 | 0.99 | 0.82 | 3.71 | 3.83 | 3.72 | 3.65 | 3.84 | 3.85 | 3.19 | 3.12 | 2.96 | 3.02 | 2.99±1.01 | 2.96±1.07 |
| Arg | 0.98 | 1.03 | 1.31 | 1.53 | 1.96 | 2.08 | 1.73 | 1.73 | 0.95 | 1.32 | 1.41 | 1.35 | 1.88 | 1.76 | 1.46±0.41 | 1.54±0.35 |
| Monomethyl-Lys | 3.33 | 3.46 | 2.91 | 3.0 | 4.04 | 4.07 | 3.21 | 3.26 | 2.36 | 2.28 | 4.00 | 3.98 | 2.25 | 2.36 | 3.16±0.71 | 3.20±0.71 |
| Carboxymethyl-Cys | 9.87 | 9.45 | 27.3 | 23.4 | 21.3 | 22.9 | 8.99 | 9.53 | 9.57 | 9.6 | 13.7 | 14.0 | 15.9 | 17.7 | 15.2±6.89 | 15.2±6.21 |
| Carboxyethyl-Lys | 23.8 | 18.8 | 6.36 | 6.49 | 18.4 | 18.3 | 1.90 | 2.07 | 5.21 | 5.05 | 3.69 | 3.62 | 7.66 | 7.97 | 9.58±8.24 | 8.91±6.88 |
| Carboxymethyl-Lys | 3.16 | 3.27 | 2.68 | 2.55 | 4.02 | 3.92 | 3.06 | 3.14 | 2.58 | 2.59 | 5.04 | 5.05 | 2.54 | 2.46 | 3.30±0.92 | 3.28±0.93 |
| ADMA | 4.53 | 4.55 | 2.43 | 2.35 | 1.51 | 1.36 | 1.82 | 1.71 | 2.93 | 2.88 | 2.11 | 1.95 | 1.39 | 1.59 | 2.39±1.08 | 2.34±1.10 |
| Carboxyethyl-Cys | 4.71 | 4.68 | 20.5 | 24.4 | 18.5 | 18.3 | 35.6 | 33.1 | 4.9 | 5.11 | 3.23 | 3.07 | 10.0 | 10.2 | 13.9±11.7 | 14.1±11.5 |
| Succinyl-Cys | 4.93 | 4.77 | 3.12 | 3.14 | 5.34 | 5.57 | 17.5 | 19.6 | 16.2 | 17.7 | 13.1 | 12.7 | 15.2 | 15.2 | 10.7±6.09 | 11.2±6.69 |
| Carboxyethyl-Arg | 10.9 | 11.6 | 4.74 | 4.82 | 50.2 | 13.4 | 23.8 | 23.1 | 8.07 | 7.43 | 2.74 | 2.74 | 3.76 | 3.86 | 14.9±17.1 | 9.56±7.18 |
| Furosine | 1.84 | 1.63 | 1.39 | 1.37 | 13.9 | 12.6 | 4.15 | 4.32 | 1.92 | 1.77 | 6.89 | 7.02 | 2.73 | 2.81 | 4.69±4.47 | 4.50±4.09 |
| Carboxymethyl-Arg | 37.9 | 29.0 | 15.6 | 15.6 | 20.2 | 19.5 | 7.87 | 7.7 | 5.83 | 5.59 | 14.1 | 13.5 | 5.90 | 5.98 | 15.3±11.3 | 13.8±38.5 |
